# The *Drosophila* maternal-effect gene *abnormal oocyte* (*ao*) does not repress histone gene expression

**DOI:** 10.1093/genetics/iyag036

**Published:** 2026-02-05

**Authors:** Risa Takenaka, Eric H Albanese, Sierra M Simmerman, Shilpi Verghese, Mandalay A E Maddox, Aida Flor de la Cruz, Janet M Young, Casey A Schmidt, Leila E Rieder, Harmit S Malik

**Affiliations:** Molecular and Cellular Biology Graduate Program, University of Washington, Seattle, WA 98195, United States; Division of Basic Sciences, Fred Hutchinson Cancer Center, Seattle, WA 98109, United States; Department of Biology, Emory University, Atlanta, GA 30322, United States; Division of Basic Sciences, Fred Hutchinson Cancer Center, Seattle, WA 98109, United States; Department of Biology, Emory University, Atlanta, GA 30322, United States; Biology Department, Lafayette College, Easton, PA 18042, United States; Division of Basic Sciences, Fred Hutchinson Cancer Center, Seattle, WA 98109, United States; Howard Hughes Medical Institute, Fred Hutchinson Cancer Center, Seattle, WA 98109, United States; Division of Basic Sciences, Fred Hutchinson Cancer Center, Seattle, WA 98109, United States; Department of Biology, Emory University, Atlanta, GA 30322, United States; Biology Department, Lafayette College, Easton, PA 18042, United States; Department of Biology, Emory University, Atlanta, GA 30322, United States; Division of Basic Sciences, Fred Hutchinson Cancer Center, Seattle, WA 98109, United States; Howard Hughes Medical Institute, Fred Hutchinson Cancer Center, Seattle, WA 98109, United States

**Keywords:** heterochromatin, histone locus body, DET1, embryonic lethality, abnormal oocyte

## Abstract

The *abnormal oocyte* (*ao*) gene of *Drosophila melanogaster* is a maternal-effect lethal gene previously identified as encoding a transcriptional regulator of core histones. However, background genetic mutations in existing *ao* mutant strains could compromise their utility in manipulating histone levels. To distinguish the true *ao* phenotype from background effects, we created 2 new *ao* reagents: a CRISPR/Cas9-mediated knockout of the *ao* allele for genetic and molecular analyses and an epitope-tagged *ao* allele for cytological experiments. Using these reagents, we confirm previous findings that loss of *ao* causes maternal-effect lethality, which can be rescued by either a decrease in the histone gene copy number or by Y chromosome heterochromatin. Our data indicate that *ao* genetically interacts with the heterochromatin, as previously suggested. However, contrary to a prior study, we detected neither Ao localization to histone genes nor *ao* repression of core histone transcript levels. Thus, the molecular basis for *ao*-associated maternal-effect lethality remains unknown.

## Introduction

In 1965, Larry Sandler and colleagues collected flies from *Drosophila melanogaster* populations near Rome, Italy, to screen for recessive mutations affecting meiosis. One of the isolated mutants produced excess female offspring when mated to males carrying an attached X^Y chromosome (Sandler et al. 1968; Sandler 1970). Sandler named this mutant *abnormal oocyte* (*abo*, recently renamed *ao*) for its aberrant sex-ratio phenotype (Sandler 1970). Subsequent analyses by Sandler showed that *ao* was one of 5 maternal-effect, embryonic semi-lethal genes located on the left arm of the second chromosome of *D. melanogaster* (Sandler 1977). These 5 genes shared the unusual property that offspring survival from homozygous-mutant mothers was directly affected by the amount of heterochromatin on X and Y chromosomes in the zygote (Sandler 1977). These genes promised to reveal the mechanistic basis of genetic interactions between euchromatin, heterochromatin, and embryonic viability.

Research on *ao* in the following 2 decades bolstered Sandler's initial observation that the viability of offspring from *ao* mutant mothers could be rescued by increasing the dosage of certain heterochromatic regions on the X, Y, and second chromosomes (Parry and Sandler 1974; Sandler 1977; Haemer 1978; Yedvobnick et al. 1980; Pimpinelli et al. 1985; Tomkiel et al. 1991). These regions, located on the distal heterochromatin on the X, the long and short arms of the Y (the *Drosophila* Y chromosome is entirely heterochromatic), and the centromeric heterochromatin on the right arm of the second chromosome, were named AO heterochromatic elements (Pimpinelli et al. 1985).

The mechanistic relationship between the *ao* mutation and AO heterochromatin remained unclear. Sandler initially hypothesized that an increase in the ribosomal DNA (rDNA) copy number (ie, the number of rDNA repeats at the locus) was responsible for AO heterochromatin's amelioration of the maternal-effect lethality. He based this hypothesis on the X- and Y-chromosomal location of the rDNA locus in *D. melanogaster* and the fact that *ao*-associated maternal-effect lethality was lower at 19.5 °C (where flies develop more slowly) than at 25.5 °C. Indeed, a subsequent study found that *ao* flies maintained as homozygous mutants developed an expansion of the rDNA locus, which alleviated the maternal-effect lethality (Krider and Levine 1975). Later studies observed the same suppressor phenotype in *ao* flies kept as homozygous stocks. However, while some found additional evidence implicating rDNA (Krider et al. 1979; Graziani et al. 1981), others disputed that rDNA copy number was the cause of the suppressor phenotype (Yedvobnick et al. 1980; Pimpinelli et al. 1985; Sullivan and Pimpinelli 1986; Cavaliere et al. 1991). Furthermore, it remained unclear whether AO heterochromatin rescues the *ao* mutation directly (ie, both *ao* and AO produce the same product) or indirectly (ie, AO produces a different product than *ao* but performs a rescue function).

Despite over 2 decades of research, it was not until 1995 that the *ao* mutation was mapped to the cytogenetic locus 32C on the second chromosome of *D. melanogaster* (Tomkiel et al. 1995). The genetic unmasking of *ao* took advantage of 2 *ao* mutants (*ao^1^*, the strain isolated from the Roman fruit-market flies, and *ao^2^*, a *P-*element-induced allele) and a transgenic rescue construct (Tomkiel et al. 1995). In 2001, the identity of *ao* was revealed as the gene *CG6093* (Berloco et al. 2001), which is the *D. melanogaster* ortholog of the *de-etiolated* or *DET1* gene, first characterized in *Arabidopsis thaliana* but later shown to be present as single-copy orthologs in most plant and animal genomes (Chory et al. 1989; Berloco et al. 2001).

This study also proposed a molecular mechanism underlying *ao*'s maternal-effect lethality (Berloco et al. 2001). It showed that *ao* encoded a protein that localized to the core histone gene promoters. Moreover, it demonstrated that *ao^1^*/*ao^2^* trans-heterozygous females produce eggs with significantly increased histone expression levels, and that reducing the histone gene copy number in *ao^1^*-homozygous females partially ameliorated the *ao-*associated maternal-effect lethality (Berloco et al. 2001). Together, these findings led to the conclusion that excess histone production in *ao* mutants caused the maternal-effect lethal phenotype. These results also suggested that heterochromatin could act as a “sink” for excess histones, explaining why excess AO heterochromatin could alleviate the embryonic lethality associated with the loss of maternal *ao*. Thus, this landmark study linked the function of *ao*, a euchromatic gene that apparently controlled histone gene expression, to heterochromatin content and, ultimately, embryonic viability.

In *D. melanogaster*, histone genes are arranged in a tandemly repeated, multigene array of approximately 100 units, each comprising all 4 core histones (H2A, H2B, H3, and H4) and the linker histone (H1), on the second chromosome. Thus, on average, a diploid *D. melanogaster* genome encodes 200 such units, even though histone copy number varies significantly within *D. melanogaster* strains and between *Drosophila* species (Lifton et al. 1978; Strausbaugh and Weinberg 1982; Kremer and Hennig 1990; McKay et al. 2015; Shukla et al. 2025). Although recently developed transgenic tools allow for more facile manipulation of histone gene copy numbers in vivo (Cook et al. 2012; McKay et al. 2015; Zhang et al. 2019), the tight regulation of histone expression still makes manipulating histone expression levels in *Drosophila* challenging. For example, flies carrying 24 copies of the histone genes have nearly identical levels of histone transcripts and proteins as wild-type flies carrying 200 copies of the histone genes, likely due to a feedback-based compensation mechanism that ensures adequate histone expression levels regardless of histone gene copy number (McKay et al. 2015). Thus, in addition to its exciting biology, *ao* emerged as a promising tool for manipulating histone gene expression in *D. melanogaster* (Chari et al. 2019).

Existing *ao* reagents, however, carry several caveats. First, only the *ao^1^* strain is still available, whereas the *ao^2^* strain has been lost. Second, the 2 mutants exhibit different phenotypes: *ao^1^* is viable as a homozygous mutant, but *ao^2^* was reported to be lethal as a homozygote (Tomkiel et al. 1995). Furthermore, although both *ao^1^/ao^1^* and *ao^1^/ao^2^* mutants exhibited maternal-effect lethality, more embryos from *ao^1^/ao^1^* mothers died at earlier stages compared to those from *ao^1^/ao^2^* mothers (Tomkiel et al. 1995). Finally, *ao^1^/ao^1^* stocks are unstable and can rapidly acquire genetic suppressors that alleviate the maternal-effect lethality (Krider and Levine 1975; Graziani et al. 1981; Manzi et al. 1986). These observations implied that genetic background effects could dramatically affect the severity of the phenotype associated with loss of *ao*.

To overcome these hurdles and to accurately characterize the *ao* phenotype, we used CRISPR/Cas9-based methods to generate new *ao* reagents: a precise knockout of *ao* to enable genetic analyses and V5 epitope*-*tagged alleles of *ao* at the endogenous locus to enable cytological visualization. Using these reagents, we recapitulated several classical genetic attributes of *ao*, including its maternal-effect lethality, which is suppressed either by a reduction in histone gene copy number or by excess heterochromatin on the Y chromosome. However, we could not confirm previous reports that Ao localizes to the histone gene cluster. We also discovered that *ao* does not affect histone transcript levels. Unlike in *ao^1^/ao^2^* flies, histone levels are unaffected in ovaries from both *Δao/Δao* and *ao^1^/ao^1^* homozygous females. Thus, although *ao* genetically interacts with heterochromatin as proposed in Sandler's original hypothesis, we conclude that the molecular basis for these interactions still remains undiscovered.

## Materials and methods

### Generation of the Δ*ao* line

We used CRISPR/Cas9 to create an *ao* knockout line. To facilitate the screening of transgenic flies, we replaced the *ao* allele with *dsRed* under the *3xP3* eye-specific promoter. We chose guide RNAs with the best efficiency score and no predicted off-targets (https://www.flyrnai.org/crispr/). We cloned guide RNAs (AGCCGGGTTCTTCTTCCGAT and AGTAATGTCTTTATTTACAA) targeting the 5′ and 3′ ends of the *ao* gene (Flybase; https://flybase.org/reports/FBgn0000018 (Öztürk-Çolak et al. 2024)) into pCFD4 U6:1_U6:3tandemgRNAs (Port et al. 2014) (Addgene plasmid #49411). The repair template sequence, including homology arms spanning approximately 1 kb upstream and downstream of the *ao* coding sequence, was cloned into pDsRed-attP (Gratz et al. 2014) (Addgene plasmid #51019). To prevent guide RNAs from targeting PAM sites, we mutated the PAM sites on the repair template using the Q5 Site-Directed Mutagenesis Kit (New England Biolabs).

BestGene Inc. (Chino Hills, CA) prepared and co-injected the plasmids into BDSC 51323 (Bloomington Drosophila Stock Center) embryos expressing *vas-Cas9* on the X chromosome. Following the injection, BestGene Inc. crossed the injected flies to a *yw* strain to isolate transformants, crossed out the *Cas9* gene, and balanced the second chromosome over *CyO.* We verified the absence of *ao* and the presence of *dsRed* with PCR and Sanger sequencing (see Supplementary Table 1 for primer sequences). We extracted genomic DNA with the DNeasy Blood & Tissue Kit (Qiagen) according to the manufacturer's protocol for insect tissue, then performed PCR using the Platinum PCR SuperMix High Fidelity (Invitrogen). The penetrance of the *CyO* phenotype decreased with temperature, which made it difficult to distinguish between homozygote-null and heterozygous flies. Therefore, we rebalanced the *Δao* allele over *CyO-gfp* marked with *mini-white*, which enabled us to screen for homozygous flies based on eye pigment color. To allow for *mini-white* visualization, the *CyO-gfp* strain carries *yw* on the X chromosome. We crossed the *yw* strain from BestGene Inc. with our *yw; CyO-gfp* strain to obtain a near-isogenic strain to our *Δao* strain and used this strain as the wild-type control for all experiments with *Δao* flies.

To obtain a *Δao* strain with a heterozygous deletion of the histone gene cluster, we crossed our *Δao* flies into the BDSC 8670 strain (Cook et al. 2012), which has a heterozygous deletion on the second chromosome corresponding to the histone gene array (chromosomal locus 39D3 to 39E2). Both the *ao* allele and histone genes are located on the left arm of the second chromosome. So, after obtaining a female fly heterozygous for both *Δao* and histone deficiency, we relied on recombination to obtain a fly with *Δao* and histone deficiency on the same second chromosome. We used this fly to make the *Δao, his(df)* stock. The histone deletion is not marked, so we used PCR using the Phusion High-Fidelity DNA polymerase (New England Biolabs) to determine its presence in the founder fly (see Supplementary Table 1 for primer sequences).

### DNA isolation and Sanger sequencing

To confirm the endogenous *Δao* and *dsRed* insertion, we collected adult homozygous transgenic *Δao* flies from the heterozygous balanced stock (*y-, w-* ; *Δao, 3xP3-dsRed/CyO, twi-GAL4, UAS-GFP*) and wild-type (*y-, w-*) flies. To isolate the DNA, we collected flies in 1.5 mL microcentrifuge tubes and euthanized them at −80 °C for 10 min. We added 50 μL of squish buffer (10 mM Tris-HCl pH 8.0, 1 mM EDTA, 25 mM NaCl, and 200 μg/ml Proteinase K, with the enzyme added fresh from −20 °C storage) to each tube, finely grinding the samples with P200 micropipette tips. We incubated the samples at 37 °C for 30 min, then at 95 °C for 2 min in heating blocks. We spun the samples at 20,000 RCF (relative centrifugal force) for 7 min and transferred the supernatant containing genomic DNA to new microcentrifuge tubes. We stored the samples overnight at 4 °C. We performed 50 μL PCR reactions using Q5 High-Fidelity 2X Master Mix (New England Biolabs) with primers designed to anneal upstream and downstream of the insertion (see R2 and F1 in Supplementary Table 1 for primer sequences). We purified the PCR product with the Monarch PCR & DNA Cleanup Kit (New England Biolabs). We designed primers (Integrated DNA Technologies) to tile both the wild-type and *Δao* allele locus. Lastly, we performed Sanger sequencing (Azenta; primers listed in Supplementary Table 1) and aligned the sequencing data to the endogenous *ao* and *Δao* sequences using SnapGene 5.1.7.

### Generation of the *ao-*transgene rescue line

We used the PhiC31 integrase system to create the “rescue” line with wild-type *ao*. We cloned the *ao* coding sequence (lacking the intron) with its endogenous promoter into the pattB plasmid, which contains an *attB* site and *mini-white* marker (Bischof et al. 2013) (DGRC stock 1420). BestGene Inc. prepared and injected the plasmid into BDSC 9750 embryos that carry the VK33 *attP* landing site on the third chromosome (Venken et al. 2006). BestGene Inc. confirmed the successful integration with PCR to verify the presence of the recombined *attL* site and the absence of the original *attP* site (see Supplementary Table 1 for primer sequences). Then, the gene encoding the PhiC31 integrase was crossed out, and the third chromosome was balanced over *TM6B*.

### Generation of the V5-tagged *ao* line

We used CRISPR/Cas9 to tag *ao* at its C-terminus with a V5 epitope. We chose a guide RNA closest to the stop codon of *ao* with no off-targets (http://targetfinder.flycrispr.neuro.brown.edu/). We cloned the guide RNA (GTATAACCACAGCACAATAG) into pCFD5 (Port and Bullock 2016) (Addgene plasmid #73914). We designed a single-stranded oligonucleotide donor (ssODN) repair template containing the V5 tag (42 bp) and approximately 55 bp of sequence upstream and downstream of the insertion site. The ssODN had a mutated PAM site to prevent re-targeting.

We sent the midi-prepped (Qiagen) pCFD5 plasmid containing the guide RNA and lyophilized ssODN to GenetiVision Inc. (Houston, TX) for injection into embryos expressing *nanos*-*Cas9.* We screened for transformants using a PCR strategy, with primers that annealed upstream and downstream of the insertion site (see Supplementary Table 1 for primer sequences). We tested for insertion of the V5 tag by the presence of a 42 bp shift in band size. Finally, we confirmed the successful insertion of the intact V5 tag by Sanger sequencing.

### Fly husbandry, fertility, and viability assays

We maintained flies on the benchtop at room temperature on corn syrup/soy media made in-house at Fred Hutchinson Cancer Center (Seattle, WA) or purchased from Archon Scientific (Durham, NC). To conduct fertility assays, we used 1- to 5-d-old males and virgin females raised at room temperature. Unless otherwise noted, we paired 4 virgin females with 2 males in a vial with corn syrup/soy media and allowed them to mate for 3 d (for X^Y assays) or 1 wk (for all other assays). To prevent larval overcrowding in the vials, we flipped the parents to new vials after 3 d and discarded the parents from the new vials 4 d later. As noted for each experiment, we set up and maintained the crosses at 18, 25, or 29 °C. We counted the adult offspring (F1) until no more progeny were produced. Data for all fertility and viability assays are available in Supplementary Table 2.

We excluded crosses with no larvae in either vial from statistical analyses. Because nongenetic factors can influence fly fertility, including variation in food and ambient humidity, we compared data only within crosses set up on the same day. We used GraphPad Prism version 10.1.1 for macOS (GraphPad Software) to plot the data and conduct statistical analyses. We performed 2-tailed Mann–Whitney *U* tests to compare the offspring counts between the 2 datasets and reported the exact *P*-values. To compare the observed offspring genotype to the theoretical Mendelian offspring genotype, we used a 1-sample proportion test (http://www2.psych.purdue.edu/∼gfrancis/calculators/proportion_test_one_sample.shtml). To compare the results of X^Y crosses, we analyzed a 2 × 2 contingency table using a 2-tailed Fisher's exact test (https://www.graphpad.com/quickcalcs/contingency1/).

We performed late-stage developmental viability assays as previously described (Spring et al. 2019). Briefly, we transferred 40-50 second-instar larvae into vials containing standard molasses fly food and waited for them to complete development. We counted the number of pupal cases in each vial and the number of eclosed adults. We calculated the percentage viability at the pupal and adult stages by dividing each value by the initial number of larvae and multiplying by 100. Each vial represents a single biological replicate, and we had 5 to 8 replicates for each genotype.

### Immunofluorescence

#### Ovaries

The tagged V5-Lsm11 control stock (V5-Lsm11 pAttB10A, Lsm11^c02047^/CyO, *twi*-GAL4, UAS::GFP) was a gift from Dr. Robert Duronio (Godfrey et al. 2009). We dissected ovaries in PBS, then fixed them with 1:1 paraPBT: heptane (paraPBT = 4% paraformaldehyde in PBS + 0.1% Triton X-100) in an Eppendorf tube for 10 min at room temperature. Following three 5-min washes in PBST (PBS + 0.1% Triton X-100), we blocked the ovaries in PBST with 3% BSA for 30 min at room temperature. We incubated the ovaries in primary antibodies overnight at 4 °C. We used a guinea pig anti-Mxc antibody (gift from Dr. Robert Duronio) (White et al. 2011) at 1:5,000 and an anti-V5 Tag monoclonal antibody (Thermo R960-25) at 1:250. After three 5-min washes in PBST, we incubated the samples with secondary antibodies in PBST for 2 h at room temperature. We used the goat anti-mouse IgG Alexa Fluor 568 (Thermo A-11031) and goat anti-guinea pig IgG Alexa Fluor 488 (Thermo A-11073), both at 1:2,000. We added Hoechst stain (Invitrogen) to the samples in the last 30 min of the incubation with the secondary antibody. After three 5-min washes with PBST, we mounted the ovaries onto slides with 20 μL of SlowFade Gold Antifade Mountant with DAPI (Invitrogen), then added coverslips and sealed with nail polish.

#### Polytene chromosomes

To overexpress *ao* in larval salivary glands, we collected virgin w[1118]; P(w[ + mC] = Sgs3- GAL4.PD)TP1 (Bloomington Stock Center #6870) females and bred them to either male *y*-, *w*- flies as a control or to male (UAS- ao.ORF.3xHA.GW)ZH-86Fb flies (Fly ORF #6093) (Bischof et al. 2013) at 23 °C. Chromosomes from F1 or from V5-tagged *ao* lines were prepared as previously described (Hodkinson et al. 2024). We dissected salivary glands from third-instar larvae and fixed them after 3 washes. First, we used a 4% paraformaldehyde and 1% Triton X-100 solution in 1× phosphate-buffered saline (PBS) for 1 min. We then transferred the glands to a 4% paraformaldehyde and 3 M acetic acid solution for 2 min. Finally, we transferred the glands to a solution of 1 part lactic acid, 2 parts H_2_O, and 3 parts acetic acid for 5 min. We placed fixed glands on a coverslip and crushed them with a slide to spread the polytene chromosomes. We plunged the slides in liquid N_2_ and removed the coverslips. We stored the slides in 95% ethanol at −20 °C. Before staining, we rehydrated the slides in 1X PBS for 15 min, then permeabilized them in 1% Triton X-100 for 10 min. We blocked the glands in 0.5% BSA in 1X PBS for 1 h at room temperature. The 0.5% BSA solution was also the diluent for the antibodies. We dispensed the primary antibody solution onto a coverslip on the slide and incubated it overnight at 4 °C. The following day, we washed off the primary antibody solution with three 15-min washes in 1X PBS. Likewise, we dispensed a secondary antibody solution and placed a coverslip over the sample. We incubated these in the dark at room temperature for 2 h. Following incubation, we washed the slides 3 times for 10 min in 1× PBS, dried them, mounted them with ProLong Diamond Anti-Fade Mountant with DAPI (P36962, Thermo Fisher Scientific), sealed the coverslip to the slide with nail polish, and stored them at 4 °C. We imaged polytene chromosomes with a ZEISS Axio Scope.A1 fluorescence microscope and the others with a Keyence BZ-X810 All-in-One-Fluorescence Microscope using the “BZ-X800 Viewer” program.

### RNA extraction and quantitative reverse transcriptase PCR

We dissected Δ*ao* or *ao^1^* ovaries from 4-d-old virgin females in PBS. To collect unfertilized eggs, we allowed 3- to 7-d-old virgin females to lay on grape plates for 7 h. We transferred 4 pairs of ovaries, or 10 unfertilized eggs, into an Eppendorf tube. We homogenized the tissue in 20 μL of TRIzol (Invitrogen) using a disposable pestle and an electric homogenizer. We stored samples at −80 °C in 100 μL of TRIzol until ready for processing. We incubated the thawed samples in 1 mL of TRIzol for 5 min, then centrifuged them at 13,000 RPM for 10 min at 4 °C to separate the supernatant. We extracted the supernatant with chloroform and extracted the soluble phase with isopropanol. After a wash in 70% ethanol, we resuspended the RNA pellet in RNase-free water. We treated the samples with DNase I (Zymo Research) and then purified them with the RNA Clean & Concentrator-5 kit (Zymo Research). We quantified the purified samples using the Qubit RNA Broad Range Assay Kit (Invitrogen), then synthesized cDNA with the SuperScript III First-Strand Synthesis System using random hexamers (Invitrogen).

To perform quantitative reverse-transcriptase PCR (RT-qPCR) on Δ*ao* or *ao^1^* ovaries and Δ*ao* unfertilized eggs (Fig. 3a and 3b, Supplementary Fig. 9, Supplementary Fig. 15a), we used the PowerUp SYBR Green Master Mix for qPCR (Applied Biosystems) with approximately 10 ng of cDNA per reaction. We used the QuantStudio 3 Real-Time PCR System (Applied Biosystems) to run the RT-qPCR experiment using the *rp49* gene as a control (see Supplementary Table 1 for primer sequences). We ran each sample in technical triplicate for each primer pair and used the median value for analysis.

To measure histone transcript levels in unfertilized eggs from *ao^1^/ao^1^* homozygous mothers (Supplementary Fig. 15b), we used a different set of primers (see Supplementary Table 1) and AzuraQuant Green Fast qPCR Mix LoRox (Azura Genomics). We report a mean of 2 technical duplicates and 4 to 5 biological replicates. We used the same setup to measure *ao* transcript levels in ovaries from 3-d-old virgin *ao-V5* flies (Supplementary Fig. 13) and in ovaries from *D. melanogaster* strains with different histone copy numbers (Fig. 4b).

To measure *ao* mRNA levels after GAL4-UAS overexpression in salivary glands (Supplementary Fig. 12), we isolated RNA from dissected salivary glands of third-instar larvae (10 pairs per replicate), placed them in 100 μL TRIzol, homogenized with plastic pestles, and stored them at −80 °C. We later brought samples to 1 mL in Trizol and rotated them for 20 min. Subsequently, we added 200 μL chloroform to the tubes and shook them vigorously, then spun them down at 20,000 RCF for 20 min at 4 °C. We transferred the aqueous phase to a new tube and repeated the chloroform addition, shaking, spinning, and aqueous-phase removal. We added 500 μL isopropanol and 5 μL glycogen to the final aqueous layer, then incubated the samples at −20 °C overnight to precipitate RNA. We spun the samples at 20,000 RCF and 4 °C for 10 min, then washed the RNA pellets with 75% ethanol. We spun the samples again in a centrifuge for 5 min at 20,000 RCF. Finally, we resuspended RNA in water and stored it at −80 °C until use. For RT-qPCR, we utilized a LunaScript RNA-to-cDNA conversion kit (New England Biolabs) to prepare samples. We used the RT-qPCR primers for *ao*, *ao*-HA, and *rp49* in Supplementary Table 1. We report a mean of 2 technical duplicates and 1 to 5 biological replicates. Biological replicates in this experiment were produced from different crosses performed asynchronously. We performed qPCR from biological replicates on separate occasions.

To analyze the RT-qPCR data, we normalized gene expression to the reference gene *rp49* and calculated fold change using the 2^−ΔΔCt^ method (Livak and Schmittgen 2001). For Figures 3a and 3b and 4b, Supplementary Figures 9 and 15, we calculated ΔΔCt by subtracting the ΔCt of the paired control sample from that of the experimental sample, and used the resulting 2^−ΔΔCt^ value (fold change). We then used the 1-sample *t*-test to compare the fold change in gene expression (normalized to *rp49* expression) of the experimental genotype relative to the control genotype.

For Supplementary Figures 12 and 13, we first calculated the average 2^−ΔCt^ for the control genotype. We then calculated the normalized relative gene expression for each experimental sample by dividing the 2^−ΔCt^ of the sample by the average 2^−ΔCt^ for the control genotype. For experiments run on more than 1 plate (Supplementary Fig. 12), we compared the 2^−ΔCt^ to the average 2^−ΔCt^ for the control genotype on the same plate. We then used the Welch's *t*-test to compare the fold change in *ao* expression (normalized to *rp49* expression) of each experimental genotype relative to the control genotype.

### Sample preparation and data analysis for the ribo-depletion RNA-seq analyses

We dissected ovaries from 4-d-old Δ*ao* and isogenic *yw* virgin females for RNA-seq. We prepared biological triplicate samples for each genotype, with 4 pairs of ovaries per replicate. Each Δ*ao* replicate sample was paired with an isogenic *yw* sample, with each paired sample set collected on the same day to account for environmental factors that might affect gene expression (eg, changes in humidity, temperature, or food composition). We transferred 4 pairs of ovaries to an Eppendorf tube for each replicate and homogenized the tissue in 20 μL of TRIzol (Invitrogen) using a disposable pestle and an electric homogenizer. We then stored the samples at −80 °C in 100 μL of TRIzol until they were ready for processing. We processed all 6 samples in parallel on the same day to ensure minimal variation during processing. We incubated the thawed samples in 1 mL of TRIzol for 5 min, then centrifuged at 13,000 RPM for 10 min at 4 °C to separate the supernatant. We extracted the supernatant with chloroform, then the soluble phase with isopropanol. After washing the RNA pellet with 70% ethanol, we resuspended it in RNase-free water. We treated the samples with DNase I (Zymo Research) and purified them with the RNA Clean & Concentrator-5 kit (Zymo Research). We quantified the purified samples with the Qubit RNA Broad Range Assay Kit (Invitrogen).

For ribosomal RNA (rRNA) depletion library preparation and sequencing, we sent the RNA to GENEWIZ NGS Services from Azenta Life Sciences (South Plainfield, NJ). GENEWIZ quantified RNA samples using Qubit 4.0 Fluorometer (ThermoFisher Scientific, Waltham, MA), checked RNA integrity with 4200 TapeStation (Agilent Technologies, Palo Alto, CA), and then added ERCC (External RNA Controls Consortium) RNA Spike-In Controls mixes to the samples before library preparation. They prepared rRNA depletion sequencing library using QIAGEN FastSelect rRNA HMR Kit (Qiagen, Hilden, Germany). To prepare the RNA sequencing library, they used the NEBNext Ultra II RNA Library Preparation Kit for Illumina by following the manufacturer's recommendations (New England Biolabs, Ipswich, MA). Briefly, they fragmented enriched RNAs for 15 min at 94 °C. They subsequently synthesized first- and second-strand cDNA. They prepared cDNA fragments by end repair and 3′-end ligation of universal adapters, then added indices and enriched the library with limited-cycle PCR. They validated sequencing libraries using the Agilent Tapestation 4200 (Agilent Technologies, Palo Alto, CA), and quantified libraries using Qubit 4.0 Fluorometer (ThermoFisher Scientific, Waltham, MA) and quantitative PCR (KAPA Biosystems, Wilmington, MA). GENEWIZ multiplexed the sequencing libraries and clustered them onto a flowcell on the Illumina NovaSeq instrument according to the manufacturer's instructions. They sequenced the samples using a 2 × 150 bp paired-end (PE) configuration. They conducted image analysis and base calling using the NovaSeq Control Software. They converted raw sequence data (BCL files) generated by the Illumina NovaSeq into FASTQ files and demultiplexed them using Illumina bcl2fastq 2.20. They allowed 1 mismatch for index sequence identification.

We mapped the reads to the *D. melanogaster* dm6 genome assembly using HISAT2 (Kim et al. 2019), which aligns multiply-mapping reads to a single genomic location. We utilized the multicov subfunction in the BEDTools suite (Quinlan and Hall 2010) to count histone reads, and used R to sum the counts at all loci for each histone gene. We combined the summed histone counts with single-copy gene counts and used DESeq2 (Love et al. 2014) to detect differential gene expression between Δ*ao* and wild-type samples. Data analyses were performed using R 4.2.2 (R Core Team 2022) and Galaxy (The Galaxy Community, 2024).

### Western blotting

We dissected ovaries from 4-d-old virgin females in PBS on ice. We transferred 5 pairs of ovaries to an Eppendorf tube, added 20 μL of 2 × Laemmli Buffer (Bio-Rad Laboratories) with 200 mM DTT and 300 mM NaCl, and flash-froze the samples in liquid nitrogen. The samples were stored at −80 °C until processing. To extract protein, we thawed the samples on ice and added protease inhibitors (EDTA-free cOmplete ULTRA Tablets, Roche). Then, we hand-pestled the samples on ice using disposable pestles. After a brief spin at 4 °C to collect the samples, we boiled them at 100 °C for 10 min, then centrifuged them at maximum speed for 2 min to obtain a clean lysate.

We loaded 10 μL of protein sample per well on the Any kD Mini-PROTEAN TGX Precast Protein Gel (Bio-Rad Laboratories). We ran the gel for 90 min at 100 V in Tris/Glycine/SDS buffer, then transferred the gel to a Trans-Blot Turbo Mini 0.2 μm Nitrocellulose membrane (Bio-Rad Laboratories) using the Trans-Blot Turbo Transfer System (Bio-Rad Laboratories). We washed the membrane 3 times with PBS, blocked in Intercept (PBS) Blocking Buffer (LI-COR) for 1 h at room temperature, then probed with primary antibodies in phosphate-buffered saline with 0.1% Tween-20 (PBST) at 4 °C overnight. We used the following primary antibodies: rabbit anti-beta-tubulin (abcam 6046) at 1:1,000, mouse anti-H2B (abcam 52484) at 1:3,000, and rabbit anti-H3 (abcam 1971) at 1:4,000. Following three 10-min washes in PBST, we incubated the membrane for 1 h at room temperature with IRDye 680RD Donkey anti-Mouse IgG (LI-COR) and/or IRDye 800CW Donkey anti-Rabbit IgG 800 (LI-COR) secondary antibodies in PBST at 1:20,000. After 3 washes with PBST and a final wash in PBS, we scanned the membrane at 700 and 800 nm on the Odyssey CLx Imager (LI-COR). We quantified the blots using Image Studio v6.0 (LI-COR). We used the Manual analysis option with median background correction (border 3 for all segments). For each pair of samples, we used the “Add Rectangle” function to draw a box around the larger band, then added a box of the same size to the other band using the “Add Selection” function. We normalized H2B or H3 expression to beta-tubulin expression as a loading control, then normalized the Δ*ao* sample to the corresponding wild-type (*yw*) sample to obtain relative quantification.

## Results

### *Δao-*knockout flies have partial maternal-effect lethality

**Fig. 1.**
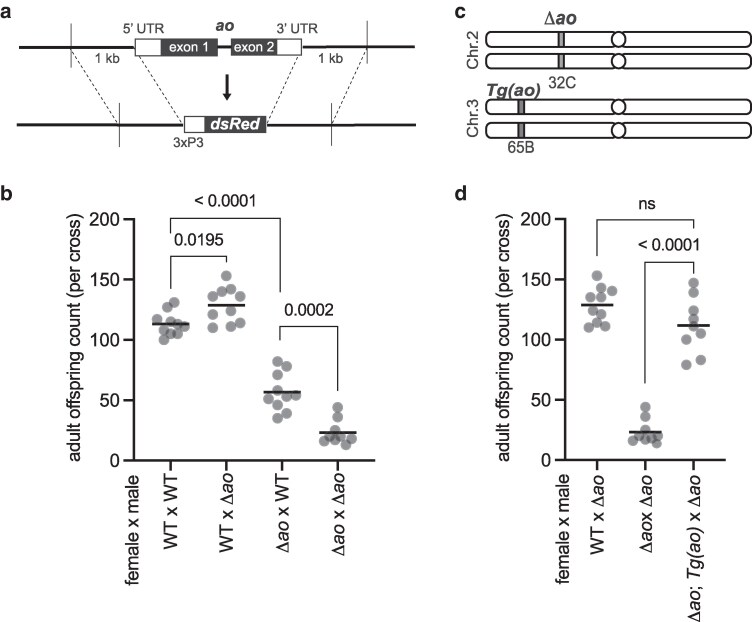
Loss of *ao* causes partial maternal-effect lethality in *D. melanogaster*. a) We replaced the *ao* coding sequence and UTRs with *dsRed* (fluorescent marker) under the control of *3xP3*, an eye-specific promoter, using CRISPR-Cas9 and homology arms (detailed in Supplementary Fig. 1). b) Crosses between Δ*ao* females and wild-type males yield fewer adult progeny than crosses between wild-type females and wild-type males or between wild-type females and Δ*ao* males, confirming that loss of *ao* leads to partial maternal-effect lethality, which is further exacerbated in crosses between Δ*ao* females and Δ*ao* males. The mild increase in offspring number in the cross between wild-type females and Δ*ao* males is not reproducible (Supplementary Fig. 3). Each point on a graph is the offspring count from a biological replicate cross performed at 29 °C. The *P-*values from 2-tailed Mann–Whitney *U* tests are shown above the compared samples. c) We integrated a “rescue” *ao* transgene construct on the *D. melanogaster* third chromosome using the PhiC31 integrase system (details in Supplementary Fig. 8). The “rescue” *ao* transgene is expressed at ∼20% of the levels of the endogenous *ao* gene (Supplementary Fig. 9). d) Despite its lower expression, the “rescue” *ao* transgene can restore the number of adult offspring produced from Δ*ao* females to nearly wild-type levels at 29 °C. The *P-*values are from 2-tailed Mann–Whitney *U* tests.

To obtain an *ao* mutant without genetic background effects, we used CRISPR/Cas9 to create a *Δao* strain using guide RNAs designed to target the start of the 5′ UTR and the end of the 3′ UTR of *ao* (Fig. 1a). To facilitate the phenotypic screening of *Δao* flies, we inserted a repair template with the fluorescent marker *dsRed* under the control of the eye-specific promoter *3xP3* using homology arms of approximately 1,000 base pairs. We verified the *ao* knockout and *dsRed* replacement, as well as the integrity of the upstream gene *ATPsynG*, using PCR and Sanger sequencing (Supplementary Figs. 1 and 2). We also generated a nearly isogenic strain to the *yw; Δao/CyO-gfp* strain except for a wild-type second chromosome in place of *Δao*, which we used as the “wild-type” control for all future experiments.

We first assessed whether our newly generated *Δao* strain recapitulated the maternal-effect lethality phenotype of *ao* mutants (Sandler 1970; Sandler 1977; Tomkiel et al. 1995). Since *ao*-associated maternal-effect lethality was more pronounced at higher temperatures (Sandler 1970), we measured the total number of adult offspring produced by *Δao* flies at 29 °C. Consistent with previous findings, we found that *Δao* females exhibit partial maternal-effect lethality when crossed to wild-type males (Fig. 1b). We found that maternal-effect lethality is exacerbated in crosses between *Δao* females and *Δao* males (Fig. 1b), confirming previous findings that a paternal copy of the wild-type *ao* allele can partially rescue zygotic survival (Pimpinelli et al. 1985; Tomkiel et al. 1995). Although our data initially suggested a slight fertility increase of *Δao* males relative to wild-type males in crosses with wild-type females (Fig. 1b), subsequent experiments revealed no significant differences in these crosses (Supplementary Fig. 3).

We confirmed our findings by measuring the survival of pupae or adults from a given number of larvae produced from *ao^1^/ao^1^* mutant mothers (Supplementary Fig. 4). This assay examines viability at later developmental stages rather than across all stages (Fig. 1b). Nevertheless, these findings (Supplementary Fig. 4) were nearly identical to our findings of offspring viability from *Δao/Δao* mothers (Fig. 1b). Thus, the maternal-effect lethality resulting from *ao* loss continues to manifest at both early and later stages of development. It can be further exacerbated by the loss of a paternal (and zygotic) *ao*.

We further assessed zygotic effects, as measured by the survival of *Δao* flies, by looking for deviations from the expected Mendelian ratio in offspring genotypes. Without a zygotic effect, the theoretical Mendelian ratio for offspring from 2 heterozygous parents should be 33% *Δao* homozygotes and 66% heterozygotes (since homozygosity for balancer chromosomes leads to lethality). In contrast to this expectation, the observed offspring genotype ratio from parents heterozygous for *Δao* was 28% homozygous and 72% heterozygous, indicating a mild but statistically significant zygotic effect (1-sample proportion test, *P =* 0.0043; Supplementary Fig. 5).

We found that heterozygous females with 1 copy of the *Δao* allele produce the same number of adult offspring as wild-type females when crossed to *Δao/Δao* males (Supplementary Fig. 6) (Sandler 1970). Finally, we found that *ao*-associated maternal-effect lethality is most pronounced at 29 °C but also manifests at 25 and 18 °C, albeit to slightly lower extents (Supplementary Fig. 7), consistent with previous findings (Sandler 1970). Thus, the *Δao* strains we created confirm previous findings from *ao^1^ and ao^2^* strains: loss of *ao* results in no significant consequences to paternal fertility but does cause maternal-effect lethality, which can be partially rescued by a wild-type paternal allele of *ao* in the zygote.

We generated a “rescue” *ao* transgene to rule out the possibility that the maternal-effect lethality in Δ*ao* strains might have resulted from an off-target mutation introduced during the CRISPR/Cas9 cleavage or repair (Fig. 1c). We used the PhiC31 integrase system (Groth et al. 2004) to insert an *ao* transgene into the third chromosome, flanked by approximately 700 base pairs upstream and 300 base pairs downstream of the *ao* protein-coding sequence. Since the *ao* promoter is poorly defined, we included the untranslated regions of neighboring genes but not their coding sequences. By crossing the *Δao* and “rescue” *ao* strains, we obtained flies with both homozygous *Δao* mutations (on the second chromosome) and 2 copies of the “rescue” *ao* transgene (on the third chromosome) (Supplementary Fig. 8). The “rescue” *ao* transgene is transcribed at only 20% of the level of the endogenous *ao* gene in ovaries (Supplementary Fig. 9, Supplementary Table 3). Despite its lower expression, the “rescue” *ao* transgene is sufficient to entirely suppress the maternal-effect lethality of *Δao* (Fig. 1d), thereby demonstrating a causal association of the maternal-effect lethality we observed with loss of *ao*.

### Ao does not detectably localize to the histone gene cluster

**Fig. 2.**
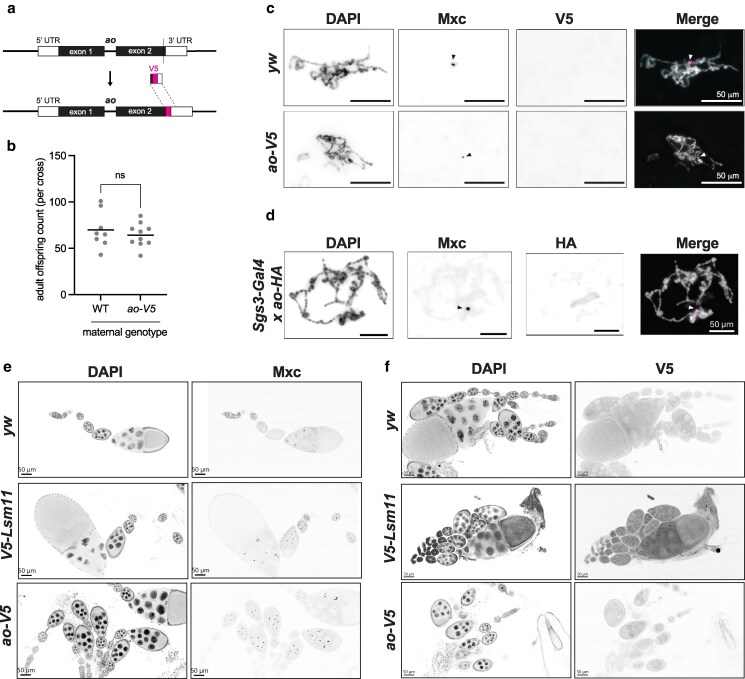
Ao-V5 does not localize to the histone gene locus in *D. melanogaster*. a) Using CRISPR/Cas9, we introduced an in-frame V5 epitope tag at the 3′ end of the *ao* coding sequence at its endogenous locus (Supplementary Fig. 10). b) Crosses between *ao-V5* females and *yw* males yield the same number of adult offspring as those between *yw* females and *yw* males at 29 °C. The *P-*values are from 2-tailed Mann–Whitney *U* tests. c) We performed immunofluorescence in polytene chromosomes from salivary glands dissected from *ao-V5* and *yw* (control) flies. We did not observe colocalization of Mxc (arrowhead), which localizes to histone locus bodies (HLBs), and Ao-V5. d) We overexpressed *ao* in salivary glands using crosses between 2 *D. melanogaster* strains, one expressing a salivary gland-specific GAL4 driver (*Sgs3-GAL4*) and the other expressing an HA-tagged *ao* transgene (*UAS-ao-HA*). Despite dramatic *ao* overexpression in salivary glands (Supplementary Fig. 12), we did not observe Ao-HA colocalizing with Mxc (arrowhead) on polytene chromosomes. e) We found that Mxc forms puncta in ovarian nurse cells in all 3 *D. melanogaster* strains assayed: *yw, V5-Lsm11,* and *ao-V5*. f) We assayed staining of the V5 epitope-tag in ovaries from 3 *D. melanogaster* strains: *yw* (which does not express a V5-tagged protein)*, V5-Lsm11* (which expresses V5-Lsm11, previously shown to localize to HLBs), and *ao-V5*. Expectedly, we observed Lsm11-V5 localizing in punctate dots in nurse cell nuclei (just like Mxc), but no V5 staining in either *yw* or *ao-V5* ovaries. In all cases, since we observed no discrete signal, we infer that epitope-tagged Ao lacks a discernible localization pattern on polytene chromosomes or in ovarian cells. (Scale bar = 50 μm).

Using a polyclonal antibody raised against the Ao protein, a previous study reported that Ao localizes to the multigene array of histone genes on the second chromosome in polytene chromosomes from salivary glands and in mitotic chromosomes from larval neuroblasts (Berloco et al. 2001). However, this antibody is no longer available. To visualize the Ao protein, we generated endogenously tagged *ao* alleles in which we tagged either the C-terminus (“*ao-V5*”) (Fig. 2a, Supplementary Fig. 10) or N-terminus (“*V5-ao*”) (Supplementary Fig. 11) of *ao* with a V5 epitope at the endogenous locus using CRISPR/Cas9. We confirmed that the *ao-V5* female flies have wild-type fertility at 29 °C, indicating that the V5 tag does not interfere with Ao protein function (Fig. 2b).

Using the V5-tagged *ao* alleles, we first sought to confirm the observation by Berloco et al. (2001) that Ao localizes to the histone gene cluster on polytene chromosomes. We stained polytene chromosomes from salivary glands with antibodies against the V5 epitope and the Multi sex combs (Mxc) protein, which localizes specifically to the histone gene cluster and is a core structural component of the histone locus body (White et al. 2011). We did not observe colocalization of Ao-V5 and Mxc using the *ao-V5* allele (Fig. 2c) or the *V5-ao* allele (Supplementary Fig. 11). Moreover, we found that Ao-V5 does not localize specifically to any chromosomal loci. Our inability to replicate prior observations (Berloco et al. 2001) could be because *ao* mRNA is nearly undetectable in salivary glands (Brown et al. 2014; Ozturk-Colak et al. 2024). We therefore overexpressed transgenic HA-tagged *ao* specifically in salivary glands using the GAL4-UAS system. Using this strategy, we observed a tremendous increase in *ao-HA* expression in salivary glands (Supplementary Fig. 12, Supplementary Table 4), yet Ao-HA still did not colocalize with Mxc at the histone genes in salivary glands (Fig. 2d) or with any distinct chromosomal loci.

Given *ao*'s maternal-effect lethal phenotype, we next investigated the expression and localization of the Ao-V5 protein in ovaries. To confirm that the V5 tag does not impair Ao expression, we first performed RT-qPCR for *ao* in ovaries. We found that *ao-*V5 homozygotes, but not heterozygotes, produce ∼1.5 times as much *ao* transcript as *yw* controls (Supplementary Fig. 13, Supplementary Table 5) without adversely affecting female fertility (Fig. 2b). We next stained ovaries for Ao-V5 and Mxc. Unfortunately, we found significant antibody cross-reactivity in this tissue, which prevented us from interpreting co-staining results. To overcome this potential artifact, we therefore separately stained for V5 and Mxc in 3 different strain backgrounds. The first is the “wild-type” *yw* strain, which lacks Ao-V5 and serves as a negative control. The second is a *V5-Lsm11* strain (Godfrey et al. 2009), which serves as a positive control, since the Lsm11 protein targets the histone locus where it is involved in the 3′ end processing of histone transcripts (White et al. 2011). The third strain is the *ao-V5* strain we have constructed. Expectedly, we find that Mxc forms clear puncta in the ovarian nurse cells in all 3 strains (Fig. 2e). Also as expected, we found no V5 staining in the *yw* strain, but clear puncta in ovarian nurse cells in the *V5-Lsm11* strain (Fig. 2f). However, we found no V5 puncta in the *ao-V5* ovaries (Fig. 2f), even though this strain expresses higher than wild-type levels of *ao* (Supplementary Fig. 13) and does not result in maternal-effect lethality (Fig. 2b). Based on our immunofluorescence experiments in multiple tissues with 2 different epitope tags, we cannot confirm prior reports of Ao localization to the histone gene cluster (Berloco et al. 2001). In addition, we found that Mxc foci are unaffected in *Δao/Δao* flies, suggesting that loss of Ao does not lead to disruption of histone locus bodies or Mxc localization (Supplementary Fig. 14).

### *Ao* does not affect histone transcript levels

**Fig. 3.**
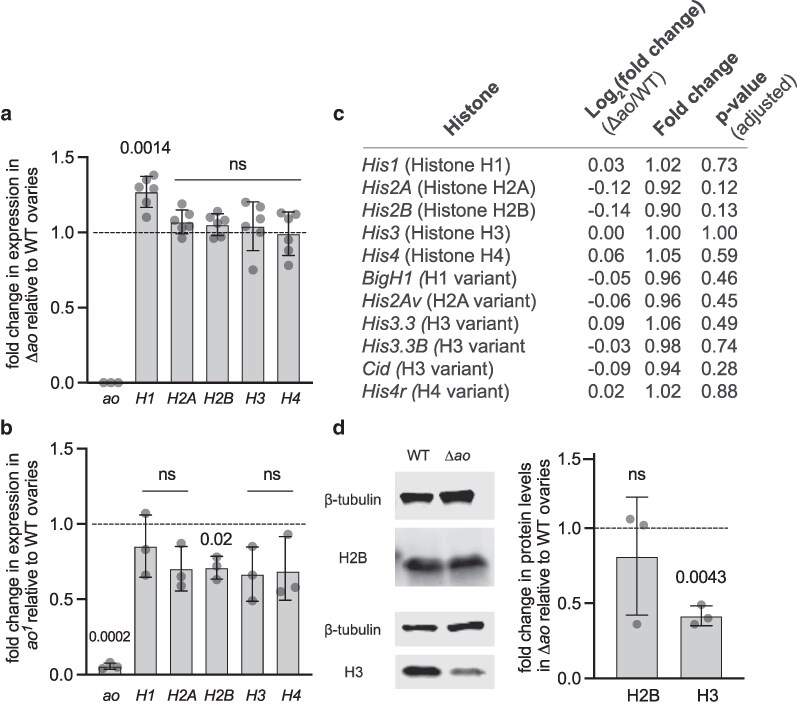
Histone expression levels in *ao* mutant ovaries. a) RT-qPCR analyses measure the levels of the linker (H1) and core (H2A, H2B, H3, H4) histone transcripts in Δ*ao* ovaries relative to ovaries from isogenic *yw* females (dashed line). Each data point is a biological replicate of 3 to 5 pairs of ovaries from 4-d-old virgin females. For each replicate, the median of the technical triplicate is shown. Gene expression has been normalized to ribosomal protein *rp49.* The *P*-values are calculated using a 1-sample *t*-test (Materials and methods). The bars represent 1 standard deviation. b) RT-qPCR on *ao^1^* ovaries presents the levels of the linker (H1) and core (H2A, H2B, H3, H4) histone transcripts relative to ovaries from *yw* females (dashed line). The *yw* strain is not isogenic to the *ao^1^* strain. Each data point is a biological replicate of 3 to 5 pairs of ovaries from 4-d-old virgin females. For each replicate, the median of the technical triplicate is shown. Gene expression has been normalized to *rp49.* The *P*-values are calculated using a 1-sample *t*-test. The bars represent 1 standard deviation. c) We performed RNA-seq on ovaries from Δ*ao* and isogenic *yw* 4-d-old virgin females. We used ribosomal RNA-depletion library preparation to also capture replication-dependent histones, whose RNA is not polyadenylated. Each genotype consisted of biological triplicates, with *n* = 4 pairs of ovaries per replicate. Log_2_(fold change) represents the differential gene expression of Δ*ao* samples relative to the wild-type samples. The adjusted *P-*values were calculated using the false discovery rate/Benjamini–Hochberg procedure. d) Western blots on Δ*ao* ovaries reveal no difference in H2B levels between Δ*ao* and wild-type (isogenic *yw*) ovaries, but 50% lower levels of H3 protein in Δ*ao* ovaries. We used beta-tubulin as a loading control for visualization and quantification. The *P*-values are calculated using a 1-sample *t*-test. The bars represent 1 standard deviation.

The same previous study also reported the remarkable finding of significant overexpression of core histone genes in *ao* mutants (Berloco et al. 2001). Based on northern blotting analyses, the study noted that unfertilized eggs from *ao^1^/ao^2^* females had a 1.6-fold (for histone H4) to 11-fold (for histone H2A) increase in core histone transcript levels relative to unfertilized eggs from wild-type Oregon-R females (Berloco et al. 2001). To quantify histone transcript levels in ovaries and unfertilized eggs from virgin females from our *Δao* strain, we performed RT-qPCR for each of the 4 core histones and the linker histone, using a similar strategy as previously reported (Bulchand et al. 2010; Rieder et al. 2017) but with slightly different primers (see Materials and methods, Supplementary Table 1). Surprisingly, we found no significant differences in core histone levels between *Δao* and isogenic wild-type samples in both ovaries and unfertilized eggs (Fig. 3a, Supplementary Table 6; Supplementary Fig. 15a, Supplementary Table 7).

Given the discrepancy between our *Δao/Δao* results and previous findings from *ao^1^/ao^2^* flies (Berloco et al. 2001), we further quantified histone transcript levels in *ao^1^/ao^1^* ovaries by RT-qPCR (the *ao^2^* stock no longer exists and cannot be assayed). Like in *Δao* ovaries, we found no evidence for a significant increase in core histone transcript levels in ovaries from *ao^1^/ao^1^* and wild-type females (Fig. 3b, Supplementary Table 8). In contrast, we observed a mild but statistically significant *decrease* in core histone H2B transcripts (Fig. 3b, Supplementary Table 8). Similarly, when assessing core histone transcript levels in unfertilized eggs from *ao^1^/ao^1^* females, we again observed either no difference or a slight decrease (for histone H2B) in core histone transcript levels (Supplementary Fig. 15b, Supplementary Table 9).

As an orthogonal method to confirm the results of the RT-qPCR assays, we also conducted RNA-sequencing to investigate whether the expression of any histone gene changed dramatically between Δ*ao* and wild-type ovaries. We used a ribosomal RNA-depletion library preparation to capture replication-dependent histones, which lack poly(A) tails (Marzluff et al. 2008). These analyses confirmed the results from our RT-qPCR analyses, showing that the expression levels of the linker, core, or variant histones were not statistically different between the 2 samples (Fig. 3c). Thus, contrary to the previously published study (Berloco et al. 2001), we conclude that loss of *ao* does not increase histone gene expression at the steady-state RNA level.

Although not explored in the original study (Berloco et al. 2001), we considered the possibility that *ao* might affect histone levels post-transcriptionally. Previous analyses found that histone H2B protein levels are approximately 2-fold higher in embryos from *ao^1^/ao^1^* females than in embryos from wild-type females, whereas H3 levels are unchanged (Chari et al. 2019). To investigate this possibility, we quantified the levels of H2B and H3 proteins in *Δao* and isogenic wild-type ovaries using western blotting analyses. We found no evidence for increased histone protein levels in *Δao* ovaries; instead, we observed a slight *decrease* in H3 protein levels (Fig. 3d, Supplementary Table 10).

Thus, our RT-qPCR, RNA-seq, and western blotting analyses show that loss of *ao* does not significantly increase histone transcript or protein levels in ovaries, challenging previous conclusions that Ao acts as a repressor of core histone expression (Berloco et al. 2001).

### Genetic interactions between *ao*, histone genes, and Y-chromosomal heterochromatin

**Fig. 4.**
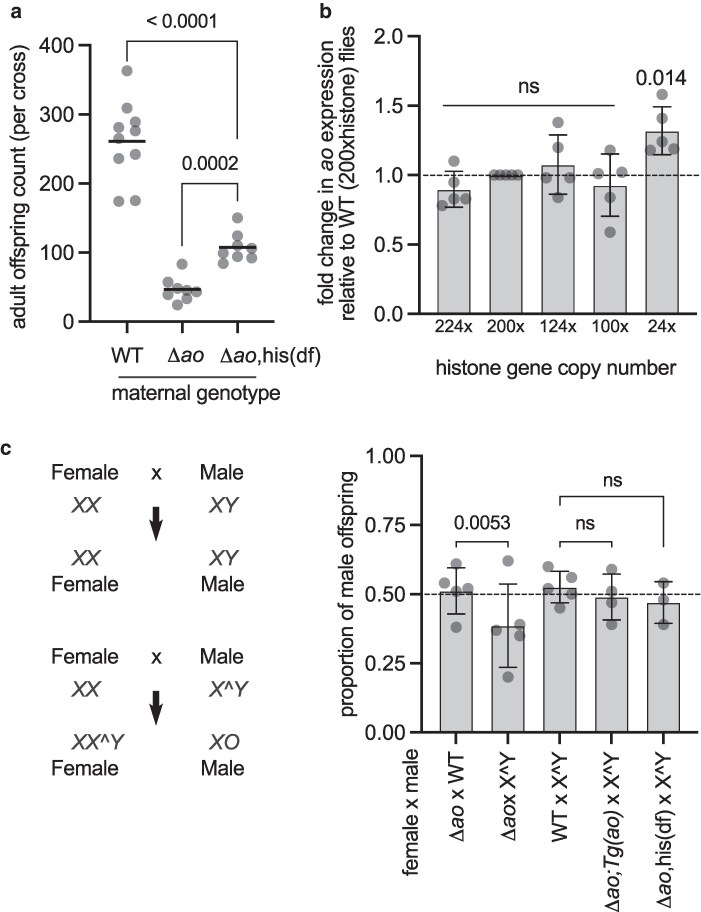
Histone deficiency and Y-chromosome heterochromatin ameliorate *ao*'s maternal-effect lethality. a) We compared the total number of offspring produced by wild-type (isogenic *yw*) females, Δ*ao* females, and Δ*ao* females harboring a heterozygous histone deficiency (*his(df),* which has a deletion from cytological locus 39D3 to 39E2, corresponding to the histone gene array; see Supplementary Fig. 16) crossed to Δ*ao* males at 29 °C. Each point on the graph is the offspring count from a biological replicate cross. The *P-*values are from 2-tailed Mann–Whitney *U* tests. b) Using RT-qPCR, we measured *ao* transcript levels in flies carrying different numbers of histone genes. Each data point is a biological replicate of 4 virgin ovaries. The mean of the biological replicate is shown for each replicate. Gene expression has been normalized to *rp49* and to the wild-type (200xhistone) case. The *P*-values are calculated using a 1-sample *t*-test (Materials and methods). The bars represent 1 standard deviation. c) Schematic of offspring produced in crosses between XX females and XY males, versus XX females and attached X^Y males. Adult offspring are produced in an equal sex ratio (50% male) in crosses between Δ*ao* females and wild-type males or crosses between wild-type females and attached X^Y males (BDSC strain 9460) at 25 °C. In contrast, in crosses between Δ*ao* females and males carrying an attached X^Y sex chromosome, adult progeny counts are skewed to produce more XX^Y females relative to XO males at 25 °C. However, this sex-ratio skew is rescued either by the presence of 2 alleles of the *ao* “rescue transgene” on the third chromosome (Fig. 1c) or by a heterozygous histone deficiency on the second chromosome (Supplementary Fig. 16). The *P-*values are from a 2 × 2 contingency table using the 2-tailed Fisher's exact test. The bars represent 1 standard deviation.

The previous finding that *ao* might encode a histone gene repressor motivated the hypothesis that *ao-*associated maternal-effect lethality results from excess histones in eggs (Berloco et al. 2001). This hypothesis led to the prediction that *ao-*associated maternal-effect lethality might be rescued by a histone deficiency in *ao-*mutant mothers. Indeed, a heterozygous deletion of the histone locus in *ao^1^*/*ao^1^* mutant females could ameliorate their maternal-effect lethality (Berloco et al. 2001). Although we found no evidence that *ao* encodes a histone gene repressor, we investigated whether a reduction in histone gene copy number could nevertheless suppress the maternal-effect lethality of *Δao* females. To test this, we crossed *Δao* flies into a strain carrying a heterozygous deletion of the histone locus (BDSC 8670) (Cook et al. 2012) to create *Δao/Δao* flies with half the number of histone genes (Supplementary Fig. 16). We found that reducing histone gene copy number partially rescues the maternal-effect lethality of *Δao* females (Fig. 4a), just as previously reported for *ao^1^*/*ao^1^* females (Berloco et al. 2001).

We also assessed the relationship between histone gene copy number and *ao* expression using flies with a deletion of the histone gene cluster (Gunesdogan et al. 2010) as well as a “12xhistone” transgene carrying only 12 copies of the histone genes inserted on the third chromosome (Fig. 4b) (McKay et al. 2015). Although wild-type diploid flies encode 200 copies of histone genes, only 12 copies (ie, flies encoding only a single 12xhistone transgene) suffice for viability (Gunesdogan et al. 2010; McKay et al. 2015). If *ao* were functioning as a histone repressor, we hypothesized that flies with such drastically reduced histone copy number could compensate by reducing *ao* expression, thereby facilitating higher histone gene expression. We used endogenous histone locus deletions and 12xhistone transgenes to generate flies carrying 224, 200 (wild-type), 124, 100, or 24 copies of the histone gene cluster (Supplementary Fig. 17). Upon quantifying *ao* transcript levels via RT-qPCR, we found that flies encoding 24 copies of histone genes had a nearly 20% increase in *ao* transcript levels compared to flies encoding 100, 124, 200 (wild-type), or 224 copies of the histone genes (Fig. 4b, Supplementary Table 11). Our findings are consistent with those of a previous study that compared the transcriptomes of 200xhistone and 12xhistone flies (McPherson et al. 2023). Although it was not the focus of the previous study, this dataset also revealed that *ao* transcript levels were slightly higher in animals with fewer copies of the histone genes. Together, these findings run counter to the expectation that *ao* negatively regulates histone gene expression.

Next, we focused on genetic interactions between *ao* and heterochromatin encoded on sex chromosomes. In crosses involving X^Y fathers, progeny either inherit the paternal attached X^Y chromosome and develop as XX^Y females, or inherit no paternal sex chromosome and develop as (sterile) XO males (Fig. 4c). Although *ao* mutant XX females produce fewer offspring than wild-type females in crosses with XY males due to their maternal-effect lethality, the sex ratio of their offspring is not skewed. However, *ao* mutant females produce an excess number of XX^Y female offspring relative to XO male offspring when crossed to males with attached X^Y chromosomes (Sandler et al. 1968; Sandler 1970, 1977; Parry and Sandler 1974). This distortion of the offspring sex ratio is attributed to the ability of specific regions of the X- and Y-heterochromatin to partially relieve *ao*-associated maternal-effect lethality (Sandler 1970, 1977; Parry and Sandler 1974; Yedvobnick et al. 1980; Pimpinelli et al. 1985; Tomkiel et al. 1991). Increased survival of female offspring with *Δao* mothers crossed to X^Y fathers would also indicate that the X^Y provides a partial rescue of the *Δao* phenotype.

We confirmed the interaction between the *ao* gene and AO heterochromatin on the Y chromosome using the *Δao* strain. Crosses between *Δao* females and wild-type males yielded nearly equal numbers of female (51%) and male (49%) offspring (Fig. 4c). In contrast, crosses between *Δao* females and attached X^Y males (BDSC 9460) yielded a 61:39 offspring ratio of females: males, significantly deviating from the 50:50 expectation (2 × 2 contingency table, 2-tailed Fisher's exact test, *P*-value = 0.0053). Normal sex ratios were restored when *Δao* females carrying 2 copies of the third chromosome “rescue” *ao* transgene were crossed to attached X^Y males (49% males). Similarly, crosses between *Δao* females carrying a histone deficiency and attached X^Y males also yielded a nearly equal sex ratio among their progeny (47% males) (Fig. 4c).

Thus, despite our finding that *ao* is not a repressor of core histone transcription, our study confirmed previous findings that *ao* loss results in maternal-effect lethality, which can be rescued either by depleting histone gene copy number or by Y chromosome-linked heterochromatin.

## Discussion

Pioneering genetic studies identified *ao* as a maternal-effect lethal gene whose phenotype depended on heterochromatin content in zygotes (Sandler et al. 1968; Sandler 1970, 1977; Parry and Sandler 1974). A satisfying explanation for *ao*'s connection to heterochromatin emerged from a molecular study 3 decades after its initial characterization, which showed that the *ao*-encoded protein localized to the histone gene cluster and that its loss led to histone overexpression in unfertilized eggs from *ao^1^/ao^2^* mothers (Berloco et al. 2001). The same study also demonstrated that reducing histone copy number ameliorated *ao*-associated maternal-effect lethality. These observations led to the model that histone overproduction in *ao/ao* mothers results in high histone levels in eggs and zygotes, which in turn leads to the maternal-effect lethal phenotype. As a result of these findings, *ao* showed promise as a potential tool for manipulating histone gene expression in *Drosophila* (Chari et al. 2019), a manipulation that cannot be achieved by simply changing histone gene copy number (McKay et al. 2015; Zhang et al. 2019).

Previous studies on *ao* function relied on reagents that have associated genetic background effects. Moreover, 2 critical reagents—the *ao^2^* allele (Tomkiel et al. 1995) and the anti-Ao antibody (Berloco et al. 2001)—are no longer available. We developed 2 novel tools to bridge this gap: a precise CRISPR/Cas9-mediated deletion of the *ao* locus (and replacement with *dsRed*) and *ao* alleles tagged with the V5 epitope at the endogenous locus. Using these reagents, we revisited key findings from the original *ao* studies. We confirmed *ao*-associated maternal-effect lethality and its amelioration via reduced histone copy number or excess Y-heterochromatin.

However, we were unable to replicate 2 important findings from the previous study (Berloco et al. 2001). First, we cannot replicate the previous finding that the Ao protein localizes to the histone gene cluster. This finding could be the result of differences between the specifically-raised, since-lost polyclonal anti-Ao antibody (Berloco et al. 2001) and the epitope tags we have used. Since we cannot detect Ao anywhere in the salivary glands (with HA and V5 tags) or ovaries (with V5 tags) despite confirming *ao* expression in both tissues, it is also possible that immunofluorescence cannot reliably detect epitope-tagged Ao in these tissues. We observed no maternal-effect lethality in flies expressing epitope-tagged *ao*-V5, indicating that the epitope tag does not interfere with *ao* function. Second, contrary to the previous study (Berloco et al. 2001), we found that *ao* is not a direct repressor of histone expression. For most histone genes, we observed no differences in histone mRNA levels between *Δao/Δao*, *ao^1^/ao^1^*, or wild-type ovaries using multiple assays. Our findings challenge the previously proposed model that histone overexpression results in *ao*'s maternal-effect lethality (Berloco et al. 2001).

Since the *ao* study was published, subsequent studies have demonstrated that dramatically reducing histone gene copy number does not decrease histone expression (McKay et al. 2015; McPherson et al. 2023). These findings also challenge the previous model, which posited that decreased histone gene expression must have compensated for histone overexpression in the absence of *ao*. Since *Δao* alleles fully recapitulate the maternal-effect lethality reported in *ao^1^/ao^1^* and *ao^1^/ao^2^* females but not the histone overexpression reported in *ao^1^/ao^2^* trans-heterozygous females, we conclude that histone overexpression is not the mechanism underlying *ao*-associated maternal-effect lethality. Our results further suggest that *ao* should not be used to manipulate histone levels. An alternative tool might be the recently described histone chaperone Nuclear Autoantigenic Sperm Protein (NASP), which encodes an H3–H4 chaperone in *Drosophila* embryos (Tirgar et al. 2023) that prevents H3 aggregation and degradation (Das et al. 2025).

Why is there a significant discrepancy in the histone overexpression phenotype between our *Δao* alleles and the previously characterized *ao^1^/ao^2^* strain? Precise deletion null alleles (like *Δao*) can often differ in phenotype from presumed null alleles that leave part of the gene intact (like *ao^1^*, which has a Doc transposon interrupting the first exon). This difference can result from transcriptional adaptation (El-Brolosy and Stainier 2017), a phenomenon in which alleles that ablate mRNA transcription entirely can be compensated for and, therefore, exhibit less severe phenotypes than those that allow the transcription of mutant mRNA (El-Brolosy et al. 2019).

In this case, however, we suspect the explanation might stem from differences in genetic background among *ao* strains. The recent discovery that histone copy number fluctuates up to 5-fold among *Drosophila* strains (Shukla et al. 2025) could partially explain the different effects of *ao* alleles on histone levels in different genetic backgrounds observed in previous studies (Berloco et al. 2001; Chari et al. 2019) and this study. Like in *Δao*/*Δao* ovaries, we did not observe a histone overexpression phenotype in *ao^1^/ao^1^* ovaries. Before our present study, *ao^1^/ao^1^* flies had never been assessed for histone-transcript levels. Indeed, the overexpression of histone mRNAs had been previously reported only for the *ao^1^/ao^2^* trans-heterozygote (Berloco et al. 2001). Unlike the *Δao* and *ao^1^* alleles, the *ao^2^* allele could not be made homozygous (Tomkiel et al. 1995), indicating the presence of 1 or more recessive lethal mutations. Moreover, whereas a wild-type *ao* transgene rescues the maternal-effect lethality of *ao^1^* (Tomkiel et al. 1995) and *Δao* (Fig. 1), a wild-type transgene rescue for histone overexpression or lethality of the *ao^1^*/*ao^2^* trans-heterozygote was never reported (Berloco et al. 2001). These observations suggest that the genetic background of the *ao^2^* allele might have conferred an additional phenotypic burden unrelated to *ao* function. For example, *P-*element insertion in the 5′ UTR of *ao* in the *ao^2^* strain might have inadvertently affected the expression of the upstream *ATPsynG* gene, which encodes an essential subunit of the mitochondrial ATP synthase (Fukuoh et al. 2014). Unfortunately, since the *ao^2^* strain is no longer available, we cannot test our hypothesis of additional background effects or how they might relate to histone overexpression.

Furthermore, Ao belongs to the DET1 family of E3 ubiquitin ligases (Berloco et al. 2001). Orthologs of *ao/DET1* are present as single-copy genes in most plant and animal genomes, including human *hDET1*, a negative regulator of a proto-oncogene (Wertz et al. 2004; Pick et al. 2007), and *Arabidopsis DET1,* which functions as a negative regulator of light-mediated growth in seedlings (Chory et al. 1989; Chory and Peto 1990; Pepper et al. 1994). *Arabidopsis* DET1 binds to nonacetylated, C-terminal H2B tails in the nucleosome (Benvenuto et al. 2002) and regulates H2B mono-ubiquitination in a light-dependent context (Nassrallah et al. 2018). Given the conservation of the plant and mammalian DET1 proteins as subunits of the COP1 Cul4A-RING E3 ubiquitin ligase complex (Wertz et al. 2004; Yanagawa et al. 2004; Bernhardt et al. 2006; Pick et al. 2007), it is highly likely that *Drosophila* Ao also functions in post-translational, rather than transcriptional, regulation. However, our western blotting analyses suggest that histones are not the post-translational target of Ao.

Our findings suggest that a molecular mechanism distinct from histone gene repression underlies *ao* function, its maternal-effect lethality, and its genetic interactions with the histone gene cluster and heterochromatin. Based on its homology and genetic interactions, we hypothesize that Ao functions as a DET1-family E3 ligase adaptor that post-translationally regulates a dosage-sensitive chromatin-associated factor, whose dosage sensitivity is buffered by both the histone locus and sex-chromosome heterochromatin. Under this model, Ao might target a component of the histone locus body (HLB), which regulates histone biogenesis (White et al. 2011; Duronio and Marzluff 2017), or a tightly associated chromatin factor, for ubiquitin-dependent degradation. In wild-type flies, Ao maintains this factor at an optimal level, allowing normal HLB assembly and histone mRNA biogenesis, and permitting embryonic development across a wide range of histone copy numbers and heterochromatin contents. However, loss of Ao would cause this factor to become overabundant or misregulated at “vulnerable” repetitive regions, disrupting early embryonic nuclear functions (eg, replication timing, chromatin packaging, chromosome segregation) and resulting in maternal-effect lethality. In this model, loss of *ao* could be partially compensated either by (1) simultaneous depletion of this unknown component via reduction or loss of the histone locus, leading to its destabilization independent of Ao, or (2) sequestration of excess levels of this factor at sex-chromosome heterochromatin sinks. An alternative possibility is that the histone gene cluster serves as a sink for another factor that becomes essential for embryonic viability in the absence of *ao*. When histone gene dosage is reduced, this factor is freed to relieve maternal-effect lethality caused by loss of *ao*. Under this model, *D. melanogaster* strains that naturally encode high histone gene copy number (Shukla et al. 2025) might be prone to more severe maternal-effect lethality upon loss of *ao*, whereas strains that naturally encode fewer genes or those carrying a histone deficiency might be better able to withstand the loss of *ao*. Although our findings challenge the previously proposed molecular model of *ao*, these possibilities highlight that understanding the basis of *ao*-associated maternal-effect lethality and its connection to histone copy number and heterochromatin remains an open and exciting question.

## Supplementary Material

iyag036_Supplementary_Data

## Data Availability

All data for the paper are available in the Supplementary materials. RNA-seq data is available in NCBI's Sequence Read Archive (SRA) under the BioProject ID of PRJNA1199081. Supplemental material available at GENETICS online.

## References

[iyag036-B1] Benvenuto G, Formiggini F, Laflamme P, Malakhov M, Bowler C. 2002. The photomorphogenesis regulator DET1 binds the amino-terminal tail of histone H2B in a nucleosome context. Curr Biol. 12:1529–1534. 10.1016/S0960-9822(02)01105-3. 12225670

[iyag036-B2] Berloco M, Fanti L, Breiling A, Orlando V, Pimpinelli S. 2001. The maternal effect gene, abnormal oocyte (abo), of *Drosophila melanogaster* encodes a specific negative regulator of histones. Proc Natl Acad Sci U S A. 98:12126–12131. 10.1073/pnas.211428798. 11593026 PMC59779

[iyag036-B3] Bernhardt A et al 2006. CUL4 associates with DDB1 and DET1 and its downregulation affects diverse aspects of development in *Arabidopsis thaliana*. Plant J. 47:591–603. 10.1111/j.1365-313X.2006.02810.x. 16792691

[iyag036-B4] Bischof J et al 2013. A versatile platform for creating a comprehensive UAS-ORFeome library in Drosophila. Development. 140:2434–2442. 10.1242/dev.088757. 23637332

[iyag036-B5] Brown JB et al 2014. Diversity and dynamics of the Drosophila transcriptome. Nature. 512:393–399. 10.1038/nature12962. 24670639 PMC4152413

[iyag036-B6] Bulchand S, Menon SD, George SE, Chia W. 2010. Muscle wasted: a novel component of the Drosophila histone locus body required for muscle integrity. J Cell Sci. 123:2697–2707. 10.1242/jcs.063172. 20647374

[iyag036-B7] Cavaliere V, Graziani F, Andone S, Manzi A, Malva C. 1991. Complete reversion of the abo phenotype in *D. melanogaster* occurs only when the blood transposon is lost from region 32E. Mol Gen Genet. 230:433–441. 10.1007/BF00280300. 1662765

[iyag036-B8] Chari S, Wilky H, Govindan J, Amodeo AA. 2019. Histone concentration regulates the cell cycle and transcription in early development. Development. 146:dev177402. 10.1242/dev.177402. 31511251 PMC7376747

[iyag036-B9] Chory J, Peto CA. 1990. Mutations in the DET1 gene affect cell-type-specific expression of light-regulated genes and chloroplast development in Arabidopsis. Proc Natl Acad Sci U S A. 87:8776–8780. 10.1073/pnas.87.22.8776. 2247447 PMC55042

[iyag036-B10] Chory J, Peto C, Feinbaum R, Pratt L, Ausubel F. 1989. Arabidopsis thaliana mutant that develops as a light-grown plant in the absence of light. Cell. 58:991–999. 10.1016/0092-8674(89)90950-1. 2776216

[iyag036-B11] Cook RK et al 2012. The generation of chromosomal deletions to provide extensive coverage and subdivision of the *Drosophila melanogaster* genome. Genome Biol. 13:1–14. 10.1186/gb-2012-13-3-r21. PMC3439972 22445104

[iyag036-B12] Das M et al 2025 NASP functions in the cytoplasm to prevent histone H3 aggregation during early embryogenesis [preprint]. bioRxiv 2025.09.03.673952. 10.1101/2025.09.03.673952 PMC13148224 42089876

[iyag036-B13] Duronio RJ, Marzluff WF. 2017. Coordinating cell cycle-regulated histone gene expression through assembly and function of the Histone Locus Body. RNA Biol. 14:726–738. 10.1080/15476286.2016.1265198. 28059623 PMC5519241

[iyag036-B14] El-Brolosy MA et al 2019. Genetic compensation triggered by mutant mRNA degradation. Nature. 568:193–197. 10.1038/s41586-019-1064-z. 30944477 PMC6707827

[iyag036-B15] El-Brolosy MA, Stainier DY. 2017. Genetic compensation: a phenomenon in search of mechanisms. PLoS Genet. 13:e1006780. 10.1371/journal.pgen.1006780. 28704371 PMC5509088

[iyag036-B16] Fukuoh A et al 2014. Screen for mitochondrial DNA copy number maintenance genes reveals essential role for ATP synthase. Mol Syst Biol. 10:734. 10.15252/msb.20145117. 24952591 PMC4265055

[iyag036-B17] Godfrey AC, White AE, Tatomer DC, Marzluff WF, Duronio RJ. 2009. The Drosophila U7 snRNP proteins Lsm10 and Lsm11 are required for histone pre-mRNA processing and play an essential role in development. RNA. 15:1661–1672. 10.1261/rna.1518009. 19620235 PMC2743060

[iyag036-B18] Gratz SJ et al 2014. Highly specific and efficient CRISPR/Cas9-catalyzed homology-directed repair in Drosophila. Genetics. 196:961–971. 10.1534/genetics.113.160713. 24478335 PMC3982687

[iyag036-B19] Graziani F et al 1981. Selective replication of ribosomal DNA repeats after loss of the abnormal oocyte phenotype in *Drosophila melanogaster*. Proc Natl Acad Sci U S A. 78:7662–7664. 10.1073/pnas.78.12.7662. 6801655 PMC349329

[iyag036-B20] Groth AC, Fish M, Nusse R, Calos MP. 2004. Construction of transgenic Drosophila by using the site-specific integrase from phage phiC31. Genetics. 166:1775–1782. 10.1093/genetics/166.4.1775. 15126397 PMC1470814

[iyag036-B21] Gunesdogan U, Jackle H, Herzig A. 2010. A genetic system to assess in vivo the functions of histones and histone modifications in higher eukaryotes. EMBO Rep. 11:772–776. 10.1038/embor.2010.124. 20814422 PMC2948182

[iyag036-B22] Haemer J . University of Washington [Dissertation] 1978.

[iyag036-B23] Hodkinson LJ et al 2024. Sequence reliance of the Drosophila context-dependent transcription factor CLAMP. Genetics. 227:iyae060. 10.1093/genetics/iyae060. 38775472 PMC11492491

[iyag036-B24] Kim D, Paggi JM, Park C, Bennett C, Salzberg SL. 2019. Graph-based genome alignment and genotyping with HISAT2 and HISAT-genotype. Nat Biotechnol. 37:907–915. 10.1038/s41587-019-0201-4. 31375807 PMC7605509

[iyag036-B25] Kremer H, Hennig W. 1990. Isolation and characterization of a *Drosophila hydei* histone DNA repeat unit. Nucleic Acids Res. 18:1573–1586. 10.1093/nar/18.6.1573. 2109309 PMC330528

[iyag036-B26] Krider HM, Levine BI. 1975. Studies on the mutation abnormal oocyte and its interaction with the ribosomal DNA of *Drosophila melanogaster*. Genetics. 81:501–513. 10.1093/genetics/81.3.501. 812775 PMC1213416

[iyag036-B27] Krider HM, Yedvobnick B, Levine BI. 1979. The effect of abo phenotypic expression on ribosomal DNA instabilities in *Drosophila melanogaster*. Genetics. 92:879–889. 10.1093/genetics/92.3.879. 119668 PMC1214043

[iyag036-B28] Lifton RP, Goldberg ML, Karp RW, Hogness DS. 1978. The organization of the histone genes in *Drosophila melanogaster*: functional and evolutionary implications. Cold Spring Harbor Symp Quant Biol. 42:1047–1051. 10.1101/sqb.1978.042.01.105. 98262

[iyag036-B29] Livak KJ, Schmittgen TD. 2001. Analysis of relative gene expression data using real-time quantitative PCR and the 2(-Delta Delta C(T)) method. Methods. 25:402–408. 10.1006/meth.2001.1262. 11846609

[iyag036-B30] Love MI, Huber W, Anders S. 2014. Moderated estimation of fold change and dispersion for RNA-seq data with DESeq2. Genome Biol. 15:550. 10.1186/s13059-014-0550-8. 25516281 PMC4302049

[iyag036-B31] Manzi A et al 1986. Changes in abo phenotypic expression without increase in rDNA in *Drosophila melanogaster*. Mol Gen Genet. 205:366–371. 10.1007/BF00430452.

[iyag036-B32] Marzluff WF, Wagner EJ, Duronio RJ. 2008. Metabolism and regulation of canonical histone mRNAs: life without a poly(A) tail. Nat Rev Genet. 9:843–854. 10.1038/nrg2438. 18927579 PMC2715827

[iyag036-B33] McKay DJ et al 2015. Interrogating the function of metazoan histones using engineered gene clusters. Dev Cell. 32:373–386. 10.1016/j.devcel.2014.12.025. 25669886 PMC4385256

[iyag036-B34] McPherson JE et al 2023. Reduced histone gene copy number disrupts Drosophila Polycomb function. Genetics. 224:iyad106. 10.1093/genetics/iyad106. 37279945 PMC10411577

[iyag036-B35] Nassrallah A et al 2018. DET1-mediated degradation of a SAGA-like deubiquitination module controls H2Bub homeostasis. eLife. 7:e37892. 10.7554/eLife.37892. 30192741 PMC6128693

[iyag036-B36] Öztürk-Çolak A et al 2024. FlyBase: updates to the Drosophila genes and genomes database. Genetics. 227:iyad211. 10.1093/genetics/iyad211. 38301657 PMC11075543

[iyag036-B37] Parry DM, Sandler L. 1974. The genetic identification of a heterochromatic segment on the X chromosome of *Drosophila melanogaster*. Genetics. 77:535–539. 10.1093/genetics/77.3.535. 4213443 PMC1213145

[iyag036-B38] Pepper A, Delaney T, Washburnt T, Poole D, Chory J. 1994. DET1, a negative regulator of light-mediated development and gene expression in Arabidopsis, encodes a novel nuclear-localized protein. Cell. 78:109–116. 10.1016/0092-8674(94)90577-0. 8033202

[iyag036-B39] Pick E et al 2007. Mammalian DET1 regulates Cul4A activity and forms stable complexes with E2 ubiquitin-conjugating enzymes. Mol Cell Biol. 27:4708–4719. 10.1128/MCB.02432-06. 17452440 PMC1951502

[iyag036-B40] Pimpinelli S, Sullivan W, Prout M, Sandler L. 1985. On biological functions mapping to the heterochromatin of *Drosophila melanogaster*. Genetics. 109:701–724. 10.1093/genetics/109.4.701. 2580754 PMC1202503

[iyag036-B41] Port F, Bullock SL. 2016. Augmenting CRISPR applications in Drosophila with tRNA-flanked sgRNAs. Nat Methods. 13:852–854. 10.1038/nmeth.3972. 27595403 PMC5215823

[iyag036-B42] Port F, Chen H-M, Lee T, Bullock SL. 2014. Optimized CRISPR/Cas tools for efficient germline and somatic genome engineering in Drosophila. Proc Natl Acad Sci U S A. 111:E2967–E2976. 10.1073/pnas.1405500111. 25002478 PMC4115528

[iyag036-B43] Quinlan AR, Hall IM. 2010. BEDTools: a flexible suite of utilities for comparing genomic features. Bioinformatics. 26:841–842. 10.1093/bioinformatics/btq033. 20110278 PMC2832824

[iyag036-B44] R Core Team . 2022. R: a language and environment for statistical computing. Vienna, Austria: Foundation for Statistical Computing. https://www.R-project.org/.

[iyag036-B45] Rieder LE et al 2017. Histone locus regulation by the Drosophila dosage compensation adaptor protein CLAMP. Genes Dev. 31:1494–1508. 10.1101/gad.300855.117. 28838946 PMC5588930

[iyag036-B46] Sandler L . 1970. The regulation of sex chromosome heterochromatic activity by an autosomal gene in *Drosophila melanogaster*. Genetics. 64:481–493. 10.1093/genetics/64.3-4.481. 17248483 PMC1212415

[iyag036-B47] Sandler L . 1977. Evidence for a set of closely linked autosomal genes that interact with sex-chromosome heterochromatin in *Drosophila melanogaster*. Genetics. 86:567–582. 10.1093/genetics/86.3.567. 408229 PMC1213695

[iyag036-B48] Sandler L, Lindsley D, Nicoletti B, Trippa G. 1968. Mutants affecting meiosis in natural populations of *Drosophila melanogaster*. Genetics. 60:525. 10.1093/genetics/60.3.525. 5728740 PMC1212059

[iyag036-B49] Shukla HG, Chakraborty M, Emerson JJ. 2025. Genetic variation in recalcitrant repetitive regions of the *Drosophila melanogaster* genome. Genome Research. 35(9):2023–2040. 10.1101/gr.280728.125. 40670076 PMC12400953

[iyag036-B50] Spring AM, Raimer AC, Hamilton CD, Schillinger MJ, Matera AG. 2019. Comprehensive modeling of spinal muscular atrophy in *Drosophila melanogaster*. Front Mol Neurosci. 12:113. 10.3389/fnmol.2019.00113. 31156382 PMC6532329

[iyag036-B51] Strausbaugh LD, Weinberg ES. 1982. Polymorphism and stability in the histone gene cluster of *Drosophila melanogaster*. Chromosoma. 85:489–505. 10.1007/BF00327345. 6290151

[iyag036-B52] Sullivan W, Pimpinelli S. 1986. The genetic factors altered in homozygous abo stocks of *Drosophila melanogaster*. Genetics. 114:885–895. 10.1093/genetics/114.3.885. 3098625 PMC1203019

[iyag036-B53] The Galaxy Community . 2024. The Galaxy platform for accessible, reproducible, and collaborative data analyses: 2024 update. Nucl Acid Res. gkae410. 10.1093/nar/gkae410. PMC11223835 38769056

[iyag036-B54] Tirgar R, Davies JP, Plate L, Nordman JT. 2023. The histone chaperone NASP maintains H3-H4 reservoirs in the early Drosophila embryo. PLoS Genet. 19:e1010682. 10.1371/journal.pgen.1010682. 36930688 PMC10058107

[iyag036-B55] Tomkiel J et al 1995. Developmental genetical analysis and molecular cloning of the abnormal oocyte gene of *Drosophila melanogaster*. Genetics. 140:615–627. 10.1093/genetics/140.2.615. 7498741 PMC1206639

[iyag036-B56] Tomkiel J, Pimpinelli S, Sandler L. 1991. Rescue from the abnormal oocyte maternal-effect lethality by ABO heterochromatin in *Drosophila melanogaster*. Genetics. 128:583–594. 10.1093/genetics/128.3.583. 1908398 PMC1204532

[iyag036-B57] Venken KJ, He Y, Hoskins RA, Bellen HJ. 2006. P [acman]: a BAC transgenic platform for targeted insertion of large DNA fragments in *D. melanogaster*. Science. 314:1747–1751. 10.1126/science.1134426. 17138868

[iyag036-B58] Wertz IE et al 2004. Human De-etiolated-1 regulates c-Jun by assembling a CUL4A ubiquitin ligase. Science. 303:1371–1374. 10.1126/science.1093549. 14739464

[iyag036-B59] White AE et al 2011. Drosophila histone locus bodies form by hierarchical recruitment of components. J Cell Biol. 193:677–694. 10.1083/jcb.201012077. 21576393 PMC3166876

[iyag036-B60] Yanagawa Y et al 2004. Arabidopsis COP10 forms a complex with DDB1 and DET1 in vivo and enhances the activity of ubiquitin conjugating enzymes. Genes Dev. 18:2172–2181. 10.1101/gad.1229504. 15342494 PMC515294

[iyag036-B61] Yedvobnick B, Krider HM, Levine BI. 1980. Analysis of the autosomal mutation abo and its interaction with the ribosomal DNA or *Drosophila melanogaster*: the role of X-chromosome heterochromatin. Genetics. 95:661–672. 10.1093/genetics/95.3.661. 6777245 PMC1214253

[iyag036-B62] Zhang W et al 2019. Probing the function of metazoan histones with a systematic library of H3 and H4 mutants. Dev Cell. 48:406–419.e5. 10.1016/j.devcel.2018.11.047. 30595536 PMC6595499

