# Supplementary material for: The Drosophila maternal-effect gene abnormal oocyte (ao) does not repress histone gene expression
Source: Genetics. 2026 Feb 5;232(4):iyag036. doi: 10.1093/genetics/iyag036 (PMC13050204; doi:10.1093/genetics/iyag036)
Supplement: iyag036_Supplementary_Data [file iyag036_supplementary_data.zip › List_of_Supplementary_Tables_GENETICS-2025-308878.docx]

**List of Supplementary Tables**

**Supplementary Table S1. List of Oligo sequences.**

**Supplementary Table S2. Details of fertility assays from all figures and supplementary figures.**

**Supplementary Table S3. RT-qPCR analyses from Fig. S9.**

**Supplementary Table S4. RT-qPCR analyses of *ao-HA* overexpression in salivary glands from Fig. S12.**

**Supplementary Table S5. RT-qPCR analyses of ao-V5 expression in ovaries from Fig. S13.**

**Supplementary Table S6. RT-qPCR analyses from Fig. 3A.**

**Supplementary Table S7. RT-qPCR analyses from Fig. S15A.**

**Supplementary Table S8. RT-qPCR analyses from Fig. 3B.**

**Supplementary Table S9. RT-qPCR analyses from Fig. S15B.**

**Supplementary Table S10. Quantification of western blot analyses from Fig. 3D.**

**Supplementary Table S11. RT-qPCR analyses from Fig. 4B.**
