# Supplementary material for: The Drosophila maternal-effect gene abnormal oocyte (ao) does not repress histone gene expression
Source: Genetics. 2026 Feb 5;232(4):iyag036. doi: 10.1093/genetics/iyag036 (PMC13050204; doi:10.1093/genetics/iyag036)
Supplement: iyag036_Supplementary_Data [file iyag036_supplementary_data.zip › Supplemental_Figure_legends_GENETICS-2025-308878.docx]

**Supplementary Figure legends**

**Supplementary Figure S1.** **CRISPR/Cas9-mediated *ao* knockout.** We replaced the *ao* coding sequence and UTRs with *dsRed* (fluorescent marker) under the control of the *3xP3* eye-specific promoter*.* The repair template sequence included homology arms spanning approximately 1kb upstream and downstream of the *ao* coding sequence. We confirmed the *ao* (1851 bp) knockout and the *3xP3-dsRed* (903 bp) replacement by PCR using two primer sets. The first primer set 1 (F1, R1) is external to the repair template. The expected size for a PCR product comprising the original wild-type *ao* gene is 4139 bp, whereas it is 3191 bp for the *dsRed* replacement (heterozygous flies have both bands). The expected wild-type band for primer set 2 (F2, R2) within the *ao* coding sequence is 883 bp. This band is absent in the homozygous knockout flies.

**Supplementary Figure S2. Sanger sequencing to confirm the knockout of *ao* and replacement with *dsRed*.** We performed Sanger sequencing to confirm the *∆ao* and *dsRed* insertion, using homozygous ∆*ao/∆ao* and isogenic *yw* adult flies. *ATPsynG*, the upstream neighboring gene, is intact in the ∆*ao/∆ao* flies. Vertical lines indicate nucleotide bases that are identical between the two genotypes.

**Supplementary Figure S3. No evidence for paternal-effect lethality in ∆*ao* flies.** We set up crosses between wildtype (Oregon R) females and wildtype (*yw*) or *Dao* males at 25°C, using three females and one male per replicate vial. The *p-*values are from two-tailed Mann-Whitney U tests.

**Supplementary Figure S4. Late-development stage viability assays for progeny from *ao^1^* females*.* (A)** We measured the percentage of larvae, resulting from crosses between *yw* (wild-type) females and males, or wild-type females and *ao^1^* males, or *ao^1^* females and wild-type males, or between *ao^1^* females and *ao^1^* males, that survive to pupation. **(B)** Similar to (A), we measured the percentage of larvae that survive to adulthood. These results can be compared to the total viability results (cumulative from all developmental stages) from *Δao* females (Fig. 1B). Comparison of survival rates between (A) and (B) indicates that there continues to be a viability effect between pupation and adulthood upon loss of *ao*.

**Supplementary Figure S5. Zygotic effects in ∆*ao* flies.** We crossed ∆*ao/CyO-GFP* heterozygous females and males to each other at 25°C*.* Since resulting *CyO-GFP/CyO-GFP* homozygous zygotes do not survive, we expect a ratio of 33% ∆*ao/*∆*ao* homozygotes among surviving adult progeny. In contrast to this expectation, we find a slightly lower recovery of ∆*ao/*∆*ao* among surviving adult progeny, indicating a mild but statistically significant zygotic effect. The *p*-value is from the one-sample proportion test.

**Supplementary Figure S6. Fertility of heterozygous ∆*ao* females.** To assess whether loss of *ao* causes a dosage-dependent maternal-effect lethality, we crossed either wildtype (isogenic *yw*), *∆ao/+* heterozygous, or *∆ao/∆ao* females to *∆ao/∆ao* males at 29°C. The total number of adult offspring produced was indistinguishable between homozygous wildtype and *∆ao/+* heterozygous female parents. The *p-*values are from two-tailed Mann-Whitney U tests.

**Supplementary Figure S7. Temperature-dependence of *ao*-associated maternal-effect lethality.** We crossed either ∆*ao* females or *∆ao/+* heterozygous females to wild-type (Oregon R) males at 18°C, 25°C, and 29°C. Each biological replicate vial had five females and two males. The p-values are from an unpaired 2-tailed Mann-Whitney U test.

**Supplementary Figure S8. Constructing an *ao* ‘rescue’ transgene.** We inserted the *ao* coding sequence and ~700bp upstream and ~300bp downstream sequence onto the 3^rd^ chromosome using the PhiC31 integrase system. Using PCR, we confirmed the absence of the endogenous *ao* allele (and its replacement with *dsRed*) on the 2^nd^ chromosome (Fig. S1), the presence of the *attL* site on the 3^rd^ chromosome, and the presence of the *ao* ‘rescue’ transgene on the 3^rd^ chromosome. The expected wildtype *ao* and knockout replacement *dsRed* bands on the 2^nd^ chromosome are 4139 bp and 3191 bp, respectively (Primer Set 1, Fig. S1). Flies carrying an *attL* site have an expected band size of 700 bp, which is present in ∆*ao* flies carrying the *ao* transgene on the 3^rd^ chromosome but missing in both wildtype and ∆*ao* flies. Wildtype flies, and ∆*ao* flies carrying the *ao* transgene on the 3^rd^ chromosome, also have a wildtype band (883 bp) for *ao,* the primers for which lie within the *ao* coding sequence (Primer Set 2, Fig. S1); this band is missing in ∆*ao* flies not carrying the *ao* transgene.

**Supplementary Figure S9. Expression levels of the *ao* ‘rescue’ transgene.** We performed RT-qPCR on ovaries from 4-day-old virgin ∆*ao;tg* homozygote (with two copies of the *ao* ‘rescue’ transgene)*,* ∆*ao*, and wildtype (isogenic *yw*) females. These experiments reveal that the *ao* rescue transgene is only expressed at 20% of the levels of the endogenous *ao* locus. Each data point is a biological replicate of 4 virgin ovaries. For each replicate, the median of the technical triplicate is shown. Gene expression has been normalized to *rp49* (data in Table S3).

**Supplementary Figure S10. Schematic of the CRISPR/Cas9-mediated insertion of the V5 tag** **on the *ao* 3' end.** We used a CRISPR/Cas9-mediated incision at the *ao* 3' end and a single-stranded oligo donor repair template containing the V5 tag (42 bp) and approximately 55 bp upstream and downstream of the insertion site to introduce the V5 tag in frame with the 3' end of the *ao* coding sequence. This insertion resulted in a 14-amino-acid V5 tag at the carboxy-terminal end of the encoded Ao protein. We confirmed the V5-tag insertion with PCR. The expected bands are 170 bp for wildtype (no V5 tag) and 212 bp with the V5 tag. We performed immunofluorescence in polytene chromosomes from salivary glands dissected from V5-*ao* flies (compare to Fig. 2c).

**Supplementary Figure S11. Schematic of the CRISPR/Cas9-mediated insertion of the V5 tag** **on the *ao* 5' end.** We used a CRISPR/Cas9-mediated incision at the *ao* 5' end and a single-stranded oligo donor repair template containing the V5 tag (42 bp) and approximately 55 bp upstream and downstream of the insertion site to introduce the V5 tag in frame with the 5' end of the *ao* coding sequence. This insertion resulted in a 14-amino-acid V5 tag at the amino-terminal end of the encoded Ao protein. We confirmed the V5-tag insertion with PCR. We investigated whether V5-Ao colocalizes with Mxc (which localizes to histone gene clusters) in polytene chromosomes of salivary glands. We did not observe colocalization of Mxc (arrowhead), which localizes to histone locus bodies (HLBs), and V5-Ao, confirming our results from Fig. 2c.

**Supplementary Figure S12. Ao-HA does not co-localize with Mxc even after overexpression in salivary glands.** Reasoning that endogenous *ao* expression is weak in salivary glands, we overexpressed Ao-HA using a cross between flies expressing a salivary gland-specific Sgs3-Gal4 driver and flies expressing UAS-driven *ao-HA*. We measured resulting *ao*-HA expression using RT-qPCR on salivary glands of 3^rd^ instar larvae produced from the following crosses: *Sgs3* female crossed to WT (*yw*) male (control cross), *UAS-ao-HA* female crossed to WT male, and *Sgs3* female crossed to *UAS-ao-HA* male. These experiments reveal that *ao* is significantly overexpressed in salivary glands of larvae from the *Sgs3 x UAS-ao-HA* cross (note that the Y-axis is on a log scale). Each data point is a biological replicate of 10 pairs of salivary glands. For each replicate, the mean of the technical duplicate is shown. Gene expression has been normalized to *rp49*. The *p-*values are from two-tailed Welch’s t-tests (data in Table S4). The bars represent one standard deviation.

**Supplementary Figure S13. *ao-V5* expression in ovaries.** We epitope-tagged *ao* in its endogenous location using the V5 epitope tag at the 3' end of the gene (Supplementary Figure S10). We then performed RT-qPCR on ovaries from 3-day-old virgin females. We found that *ao-V5* homozygotes, but not heterozygotes, produce ~1.5 times as much *ao* transcript as wildtype (*yw*) controls. Each data point is a biological replicate of ~10 pairs of ovaries. For each replicate, the mean of the technical duplicate is shown. Gene expression has been normalized to *rp49*. The *p-*values are from two-tailed Welch’s t-tests (data in Table S5). The bars represent one standard deviation.

**Supplementary Figure S14. Mxc localization to the histone locus is unaffected by the loss of Ao.** We performed immunofluorescent staining in ovaries to show that Mxc forms nuclear puncta in ovarian nurse cells from both *∆ao* and isogenic *yw* flies. Thus, Mxc localization to histone locus bodies is unaffected in *∆ao* flies.

**Supplementary Figure S15. Histone expression levels in unfertilized eggs from ∆*ao* or *ao^1^* females. (A)** We used RT-qPCR on 0-7 hour unfertilized eggs from 3-7 day old virgin ∆*ao* or isogenic *yw* females to assess levels of histone expression. We used RNA from 10 unfertilized eggs for each genotype. The median of the technical triplicate is shown. Gene expression has been normalized to *rp49.* We found no evidence for significantly elevated histone expression in ∆*ao* relative to wildtype (dashed line) except for a slightly increased histone H1 expression (consistent with Fig. 3a) (data in Table S7). **(B)** We performed RT-qPCR on unfertilized eggs from *ao^1^* females relative to *yw* females. Expectedly, *ao* expression is not detectable in *ao^1^* unfertilized eggs. The expression levels for most histones are also not significantly deviant from wildtype (dashed line). However, histone H2B levels are significantly lower, just as they are in ovaries from *ao^1^* females (Fig. 3b). The *p*-values are calculated using a one-sample t-test (data in Table S9). The bars represent one standard deviation.

**Supplementary Figure S16. Introducing a histone deficiency in a *∆ao* strain.** The endogenous *ao* and the histone loci are both encoded on the *D. melanogaster* 2^nd^ chromosome, with *ao* present on the 32C cytogenetic location and histone genes on the 39DE cytogenetic location. We used recombination in female flies heterozygous for *∆ao* and the histone deficiency to produce a fly homozygous for *∆ao* and carrying a heterozygous histone deficiency (Fig. 4a). We screened resulting flies using visual screening (*ao* is replaced by *dsRed* in *∆ao*, and the *CyO-gfp* balancer chromosome is marked with *mini-white*) followed by PCR-mediated screening to assay for the loss of the histone locus to recover flies carrying *∆ao* and the histone deficiency on the same chromosome.

**Supplementary Figure S17. Constructing *D. melanogaster* strains with different histone gene copy numbers. (A)** Typically, *D. melanogaster* strains encode core, replication-coupled histones (H2A, H2B, H3B, H4) and the linker histone H1 in a repeat unit in a multigene array. This array is repeated ~100 times at the 39DE cytological location on the 2^nd^ chromosome. Recent efforts have also introduced a 12xhistone transgene array at the 86F6 cytological locus on the 3^rd^ chromosome. By taking advantage of the presence or absence of each of these arrays, we can produce *D. melanogaster* strains encoding different histone gene copy numbers, ranging from ~224 copies (homozygous for both endogenous histone locus alleles and 12xhistone arrays) to 24 copies (homozygous deletion of both endogenous histone locus alleles but presence of two 12xhistone arrays), and several intermediate configurations of ~200, ~124, and ~100 copies. We used these histone gene configurations to investigate the relationship between histone gene copy number and *ao* expression (Fig. 4b).
