# Supplementary material for: The Drosophila maternal-effect gene abnormal oocyte (ao) does not repress histone gene expression
Source: Genetics. 2026 Feb 5;232(4):iyag036. doi: 10.1093/genetics/iyag036 (PMC13050204; doi:10.1093/genetics/iyag036)
Supplement: iyag036_Supplementary_Data [file iyag036_supplementary_data.zip › Supplemental_Figure_S2_GENETICS-2025-308878.pdf]

(| = identical between Δ*ao* and isogenic *yw* strain)

```
                                  ATP synthase subunit G
CAAGGTGGAACTGACGCCCCCGACGCCCGCCGATATTCCGGCCATTCGCCAAGGACTGGG
||||||||||||||||||||||||||||||||||||||||||||||||||||||||||||

CAACATCATCAAGGGAGCCAAGACCGGCGCCTACAAGAACCTCACGGTTCGCGAGGCCTG
||||||||||||||||||||||||||||||||||||||||||||||||||||||||||||

GCTTAACACCCTGGTGACCGCCGAGGTCATCTTCTGGTTCTACATCGGCGAGTGCATCGG
||||||||||||||||||||||||||||||||||||||||||||||||||||||||||||

CAAGCGTCACATTGTAGGCTACAATGTCTAAGCTTACTATAGTCTCCGCTTGGCAGTCAC
||||||||||||||||||||||||||||||||||||||||||||||||||||||||||||

TGGAATGGGCAACGTAATCCCTAACAGATGTGTATATTTATATGTCTGCGAACATTTCGA
||||||||||||||||||||||||||||||||||||||||||||||||||||||||||||

CTCTGAATAAAGTGAAATAGTAATTTAAAATTCCGAAAATTTTCGAAAAATACATTGTTT
||||||||||||||||||||||||||||||||||||||||||||||||||||||||||||

TTTGAAAACCGTTAGAACGTTTGCGCGGGATTTGTGTAAGCTAAAGATGAGGTGATGTAA
||||||||||||||||||||||||||||||||||||||||||||||||||||||||||||

AACCAAGTTTGGAATTAAAAGTTGACGATATTTAATGATAAAAATTAAAAAAATATATGT
||||||||||||||||||||||||||||||||||||||||||||||||||||||||||||

AATGTTATACATCAAATGTTTATGAAACGGTGTCGTAATCAAAGAGGCTAATGGTTCAGA
||||||||||||||||||||||||||||||||||||||||||||||||||||||||||||

AATACATAATATACTTAGAGCATTTAAAAGCACTCAAGAATAATTTTATTTAAAAAAAAA
||||||||||||||||||||||||||||||||||||||||||||||||||||||||||||

                                     Upstream of ao start codon
AATAAATAAATCTAAATTTGCTTTTCATAGATAATTCATTTACACATTTTTTTAAACAAA
||||||||||||||||||||||||||||||||||||||||||||||||||||||||||||

GTAAAGTAAGATGTATTGAATTATTTTATTTATAAATAACGTTTTTTATTTGAATCTTGA
||||||||||||||||||||||||||||||||||||||||||||||||||||||||||||

AAAGCATTAATATTATTATTATTATACTTATATTTTTTAACAACAAAACTTTTGTAGACA
||||||||||||||||||||||||||||||||||||||||||||||||||||||||||||

GAGTAAATTTTTGTAATCTAACTGCGGTCACACTTTACTTTAGTTACCTTTCGATCGGAA
|||||||||||||||||||||||||||||||||||||||||||||||||| |||||||||

                                                 3XP3 promoter sequence
GAAGAACCCGGCTGGATCTAATTCAATTAGAGACTAATTCAATTAGAGCTAATTCAATTA
|||||||||||||||||

GGATCCAAGCTTATCGATTTCGAACCCTCGACCGCCGGAGTATAAATAGAGGCGCTTCGT

CTACGGAGCGACAATTCAATTCAAACAAGCAAAGTGAACACGTCGCTAAGCGAAAGCTAA

GCAAATAAACAAGCGCAGCTGAACAAGCTAAACAATCGGCTCGAAGCCGGTCGCCACCAT

                                  dsRed
GGCCTCCTCCGAGGACGTCATCAAGGAGTTCATGCGCTTCAAGGTGCGCATGGAGGGCTC
```

```
CGTGAACGGCCACGAGTTCGAGATCGAGGGCGAGGGCGAGGGCCGCCCCTACGAGGGCAC

CCAGACCGCCAAGCTGAAGGTGACCAAGGGCGGCCCCCTGCCCTTCGCCTGGGACATCCT

GTCCCCCCAGTTCCAGTACGGCTCCAAGGTGTACGTGAAGCACCCCGCCGACATCCCCGA

CTACAAGAAGCTGTCCTTCCCCGAGGGCTTCAAGTGGGAGCGCGTGATGAACTTCGAGGA

CGGCGGCGTGGTGACCGTGACCCAGGACTCCTCCCTCCAGGACGGCTCCTTCATCTACAA

GGTGAAGTTCATCGGCGTGAACTTCCCCTCCGACGGCCCCGTAATGCAGAAGAAGACTAT

GGGCTGGGAGGCGTCCACCGAGCGCCTGTACCCCCGCGACGGCGTGCTGAAGGGCGAGAT

CCACAAGGCCCTGAAGCTGAAGGACGGCGGCCACTACCTGGTGGAGTTCAAGTCCATCTA

CATGGCCAAGAAGCCCGTGCAGCTGCCCGGCTACTACTACGTGGACTCCAAGCTGGACAT

CACCTCCCACAACGAGGACTACACCATCGTGGNNCAGTACGAGCGCGCCGAGGGCCGCCA

                          Downstream of ao Ter codon
CCACCTGTTCCTGTAGATCTCGTTTTACCTTTTCAGTAATGTCTTTATTTACAATGATAA
                |||||||| ||||||||||||||| ||||||||||||||| |||

GAGAACATTTCTTTGTTTTATTTAATCAAAGACTGTTAATATTCCAAGTACTGTTTAAAT
|  |||||||||||||||||||||||||||||||||||||||||||||||||||||||||

CTGACAAACACTTTTTAATTCGACTTTGCTATATTGTATTGAAGGCCCACTTCAAACTTG
||||||||||||||||||||||||||||||||||||||||||||||||||||||||||||

AGGCCCAATCCGACGTTTAAAGTTTCATGTAACATTGTATCTCGACTGCGACTACGTTGA
||||||||||||||||||||||||||||||||||||||||||||||||||||||||||||

AGATTGGTGGTGACCGTCCTCGTTGTATTCCTTGTTGACCTTAAGGGAATGATTAATAAC
||||||||||||||||||||||||||||||||||||||||||||||||||||||||||||

GCTCGAGCCGCCTGGAGGATAGTTCGGGGCAGGCCCTGANCCCGGGCCGCTGGCAGGCTG
||||||||||||||||||||||||||||||||||||||| ||||||||||||||||||||

CTGTGCCTGAGGCTGATTGGGGATCGCATTTGGGTTGTTAACTGGATTCTGTCCGGGAGC
|||||||||| |||||||||||||||||||||||||||||||||||||||||||||||||
```

Figure S2
