# Supplementary figures and images for: The Drosophila maternal-effect gene abnormal oocyte (ao) does not repress histone gene expression
Source: Genetics. 2026 Feb 5;232(4):iyag036. doi: 10.1093/genetics/iyag036 (PMC13050204; doi:10.1093/genetics/iyag036)

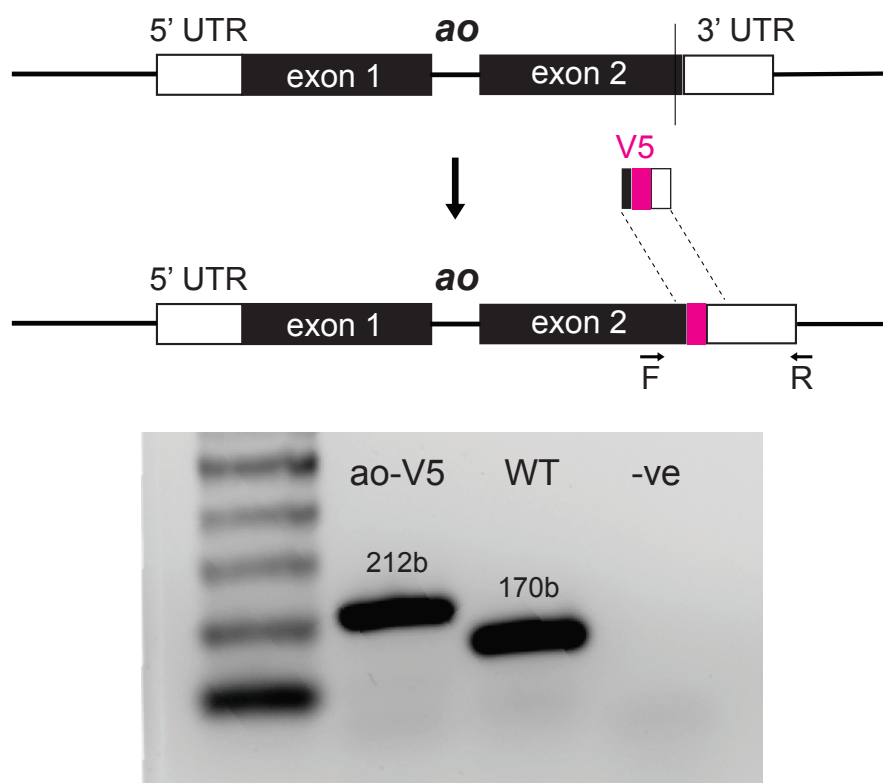

**Figure S10**

Supplement: iyag036_Supplementary_Data [file iyag036_supplementary_data.zip › Supplemental_Figure_S10_GENETICS-2025-308878.pdf]

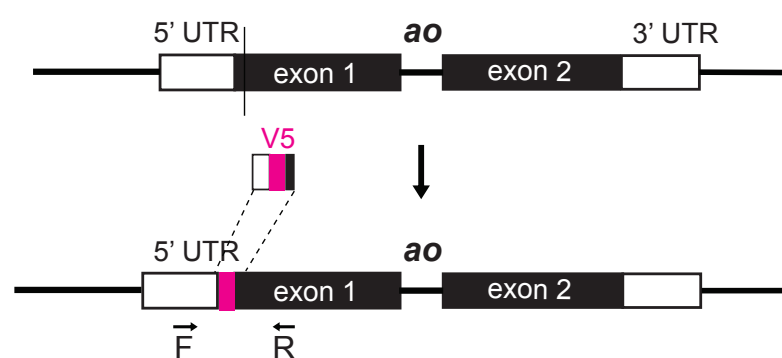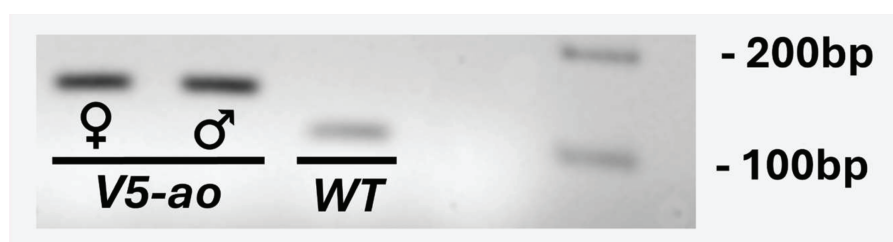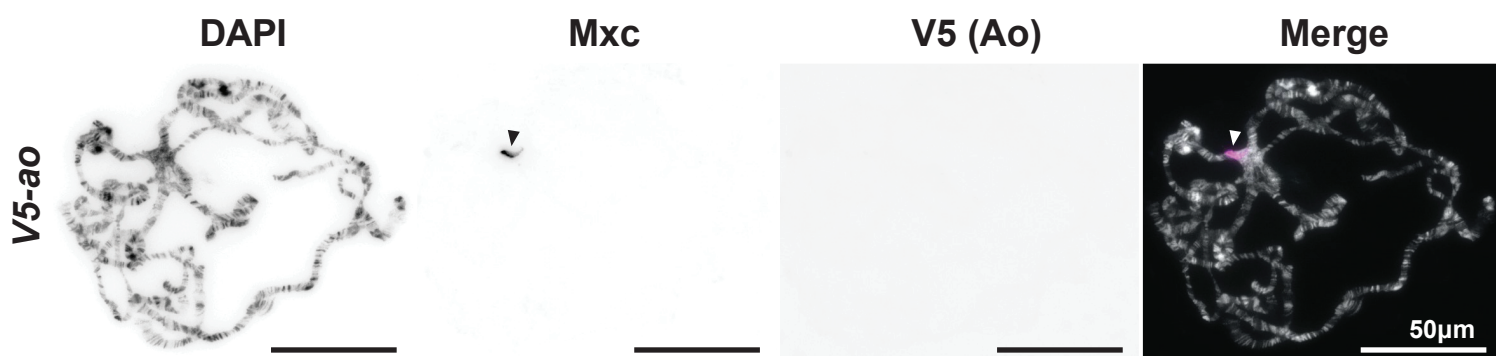

**Figure S11**

Supplement: iyag036_Supplementary_Data [file iyag036_supplementary_data.zip › Supplemental_Figure_S11_GENETICS-2025-308878.pdf]

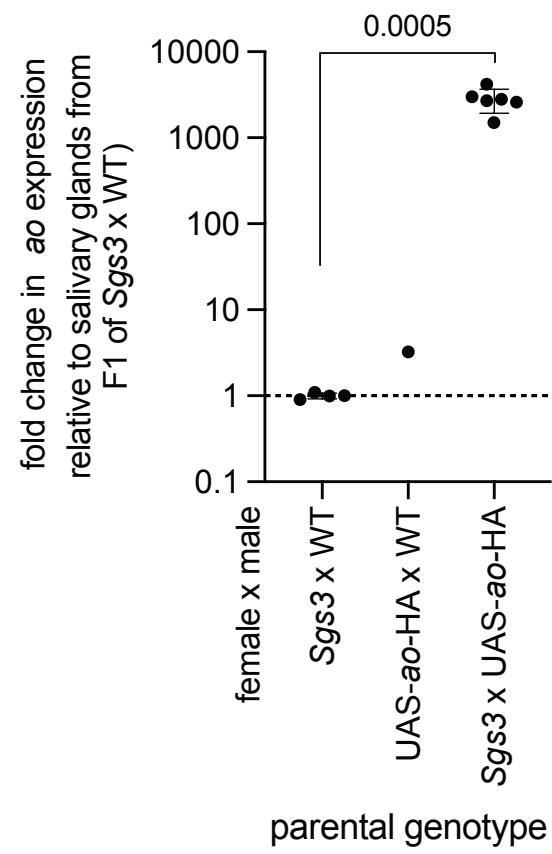

**Figure S12**

Supplement: iyag036_Supplementary_Data [file iyag036_supplementary_data.zip › Supplemental_Figure_S12_GENETICS-2025-308878.pdf]

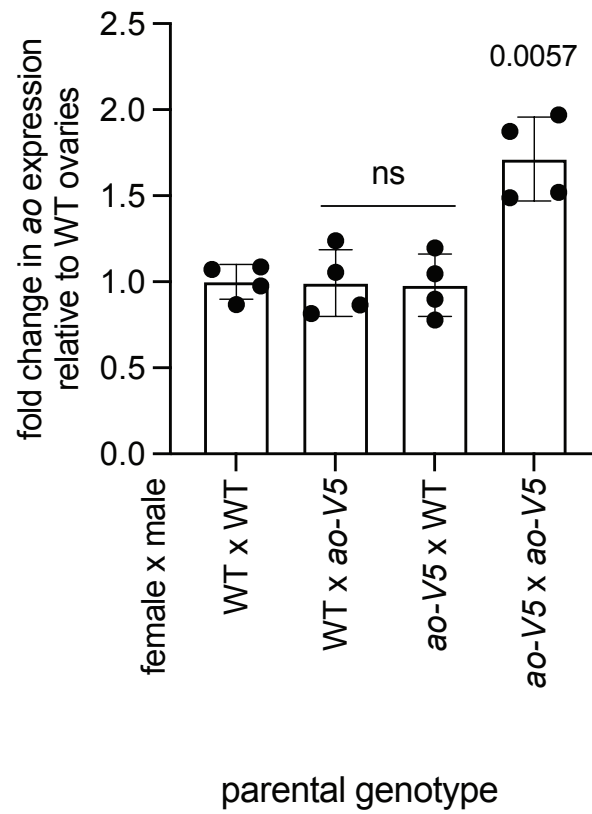

**Figure S13**

Supplement: iyag036_Supplementary_Data [file iyag036_supplementary_data.zip › Supplemental_Figure_S13_GENETICS-2025-308878.pdf]

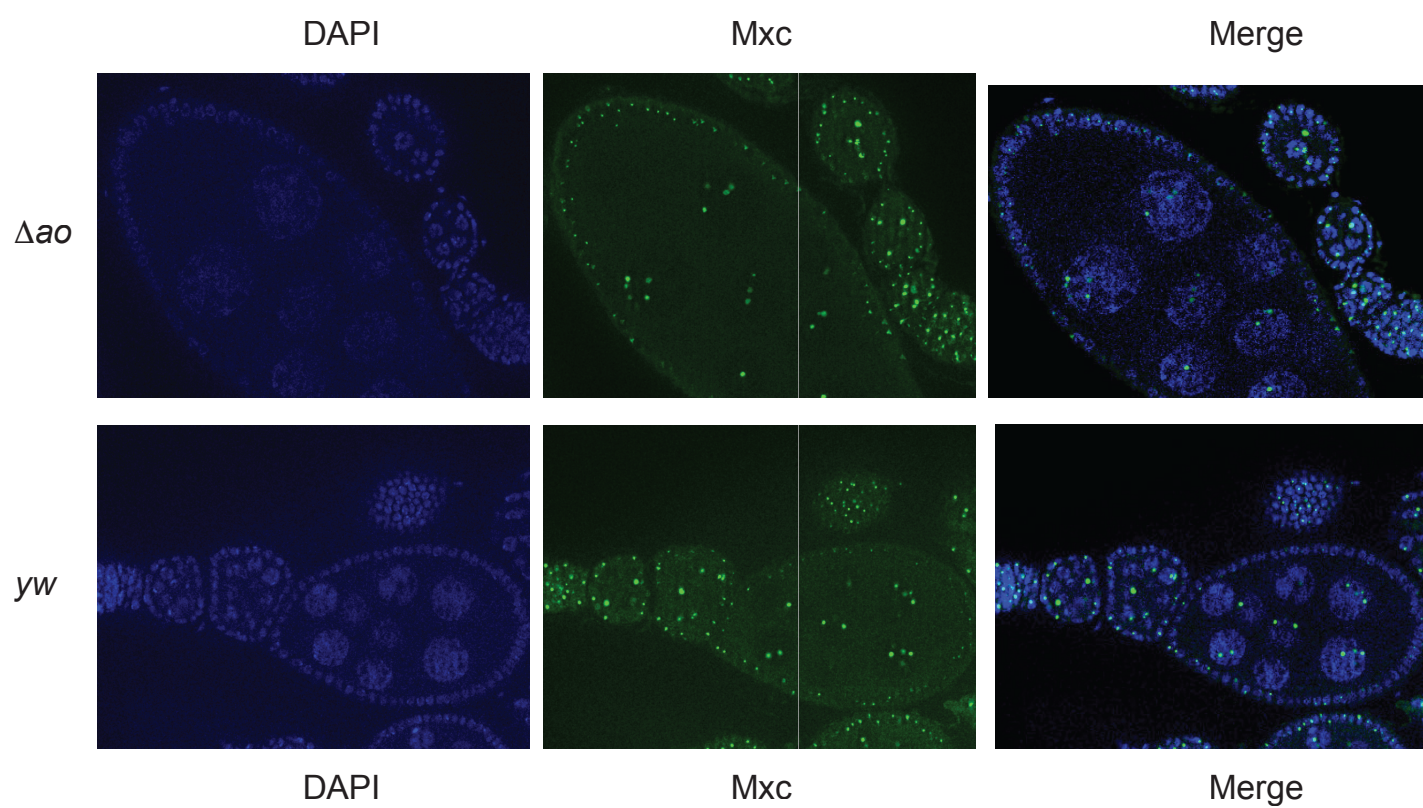

**Figure S14**

Supplement: iyag036_Supplementary_Data [file iyag036_supplementary_data.zip › Supplemental_Figure_S14_GENETICS-2025-308878.pdf]

**A**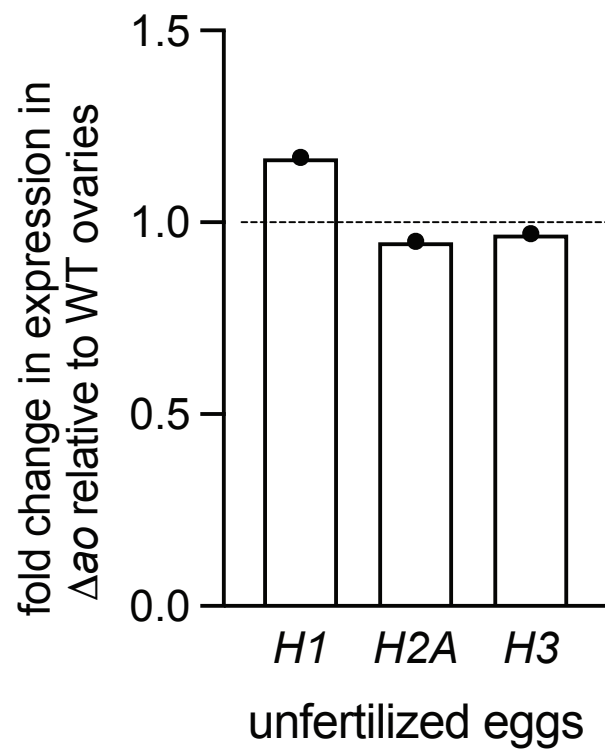**B**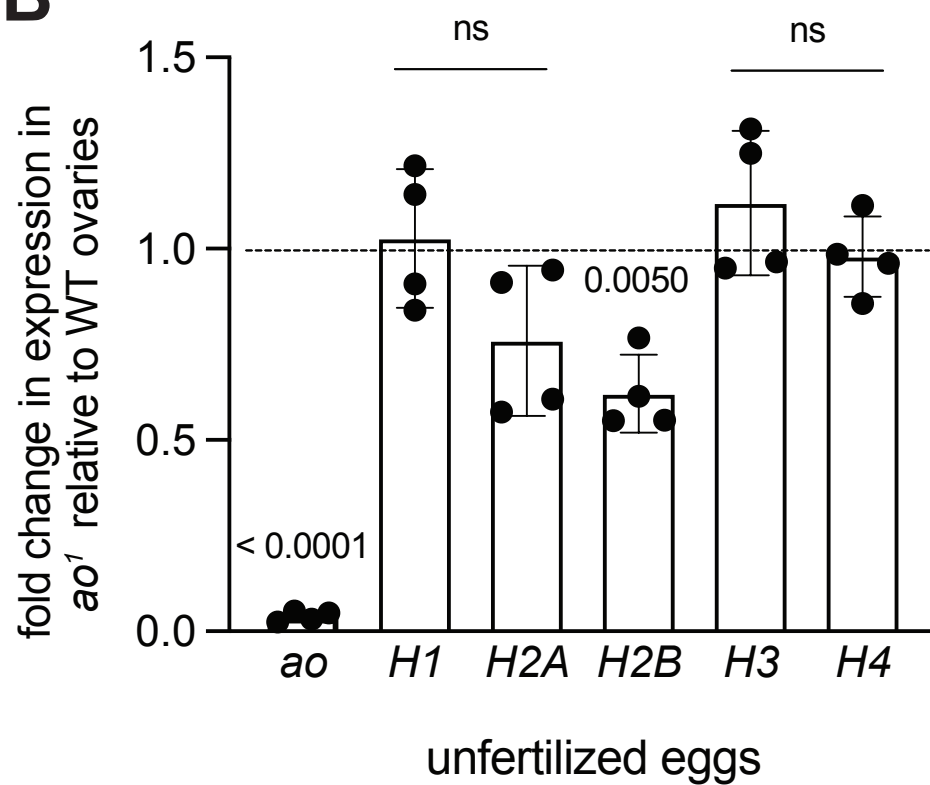**Figure S15**

Supplement: iyag036_Supplementary_Data [file iyag036_supplementary_data.zip › Supplemental_Figure_S15_GENETICS-2025-308878.pdf]

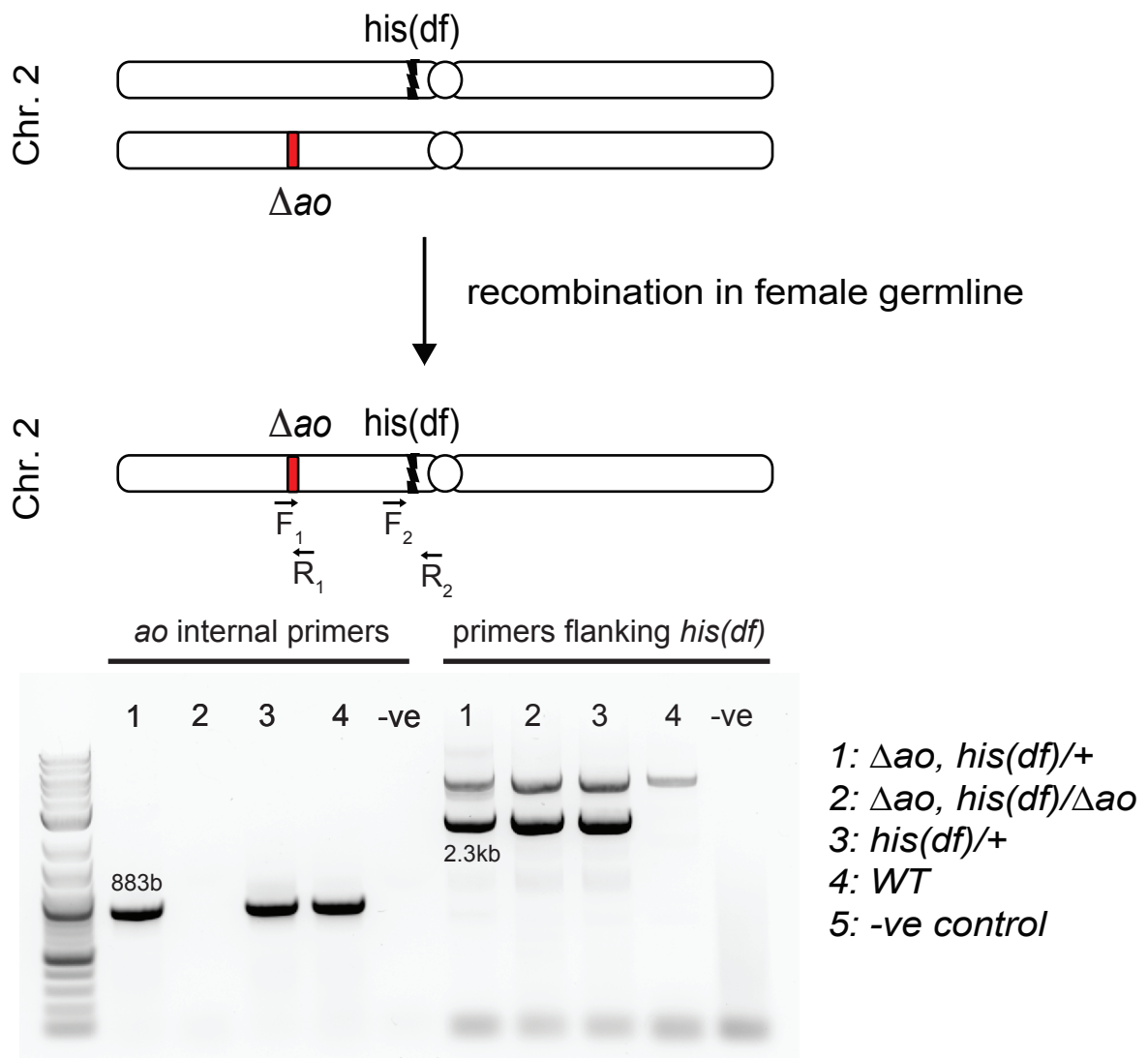

**Figure S16**

Supplement: iyag036_Supplementary_Data [file iyag036_supplementary_data.zip › Supplemental_Figure_S16_GENETICS-2025-308878.pdf]

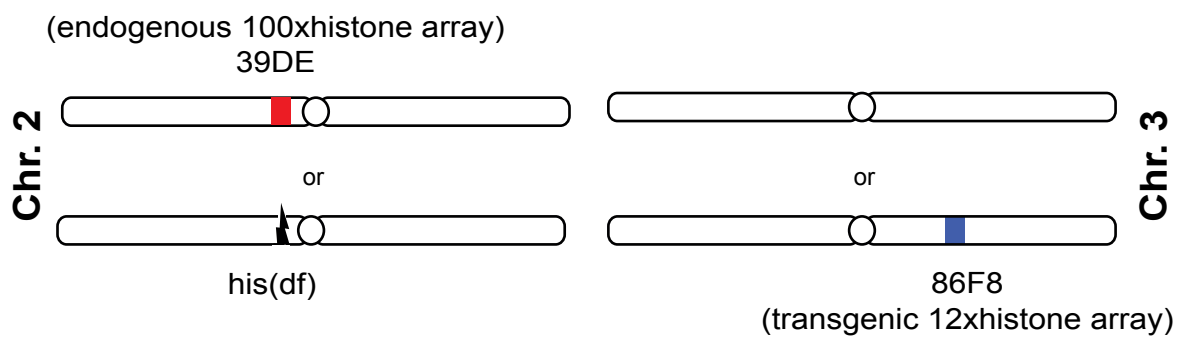

### Different histone copy number configurations

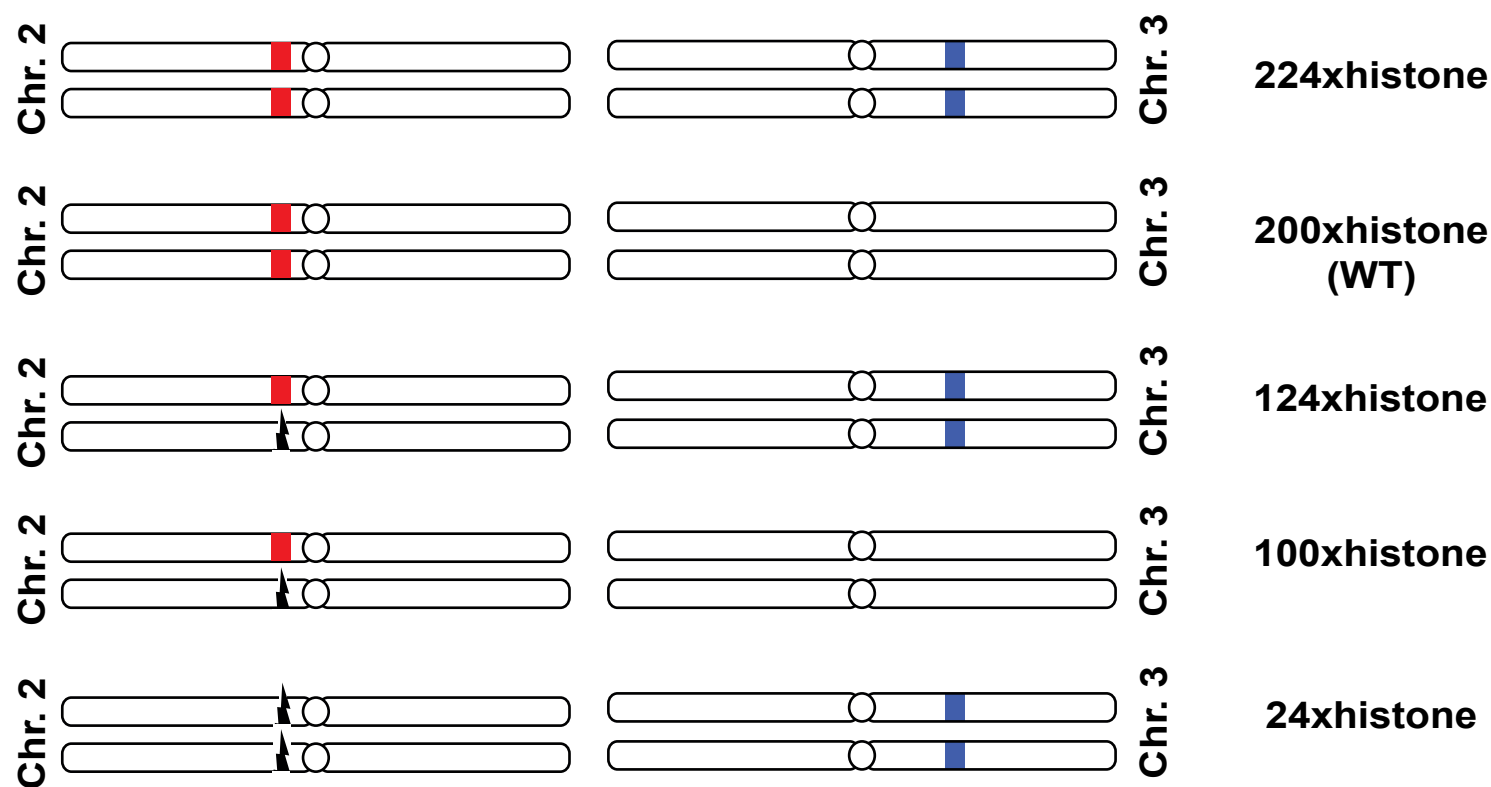

Figure S17

Supplement: iyag036_Supplementary_Data [file iyag036_supplementary_data.zip › Supplemental_Figure_S17_GENETICS-2025-308878.pdf]

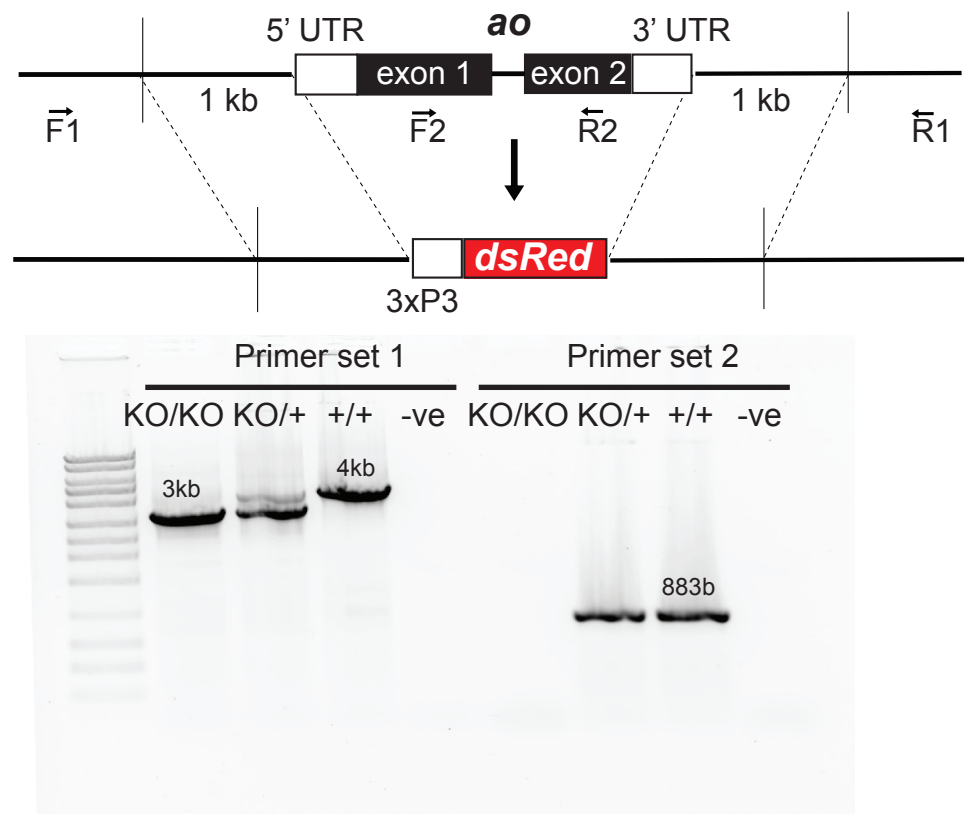

Figure S1

Supplement: iyag036_Supplementary_Data [file iyag036_supplementary_data.zip › Supplemental_Figure_S1_GENETICS-2025-308878.pdf]

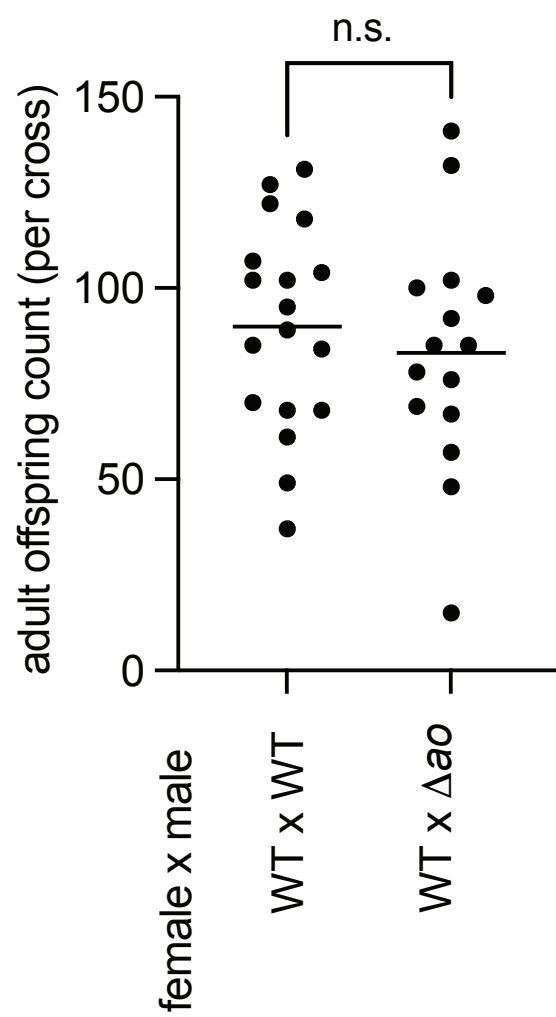

**Figure S3**

Supplement: iyag036_Supplementary_Data [file iyag036_supplementary_data.zip › Supplemental_Figure_S3_GENETICS-2025-308878.pdf]

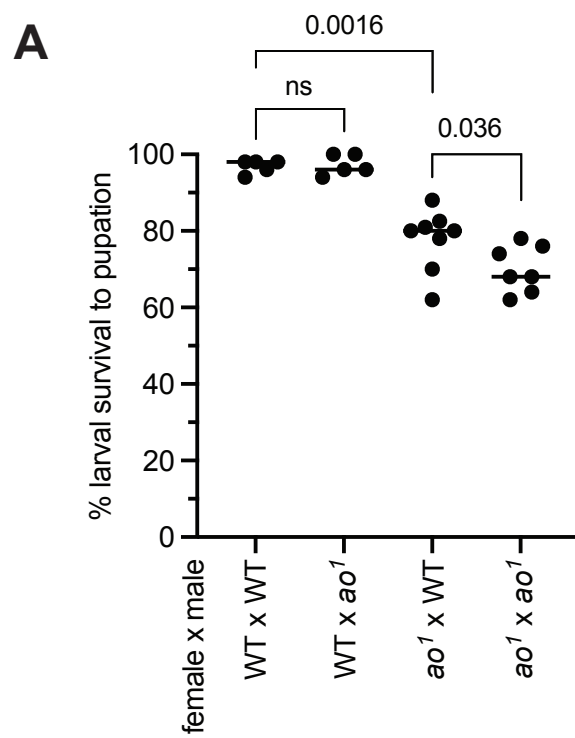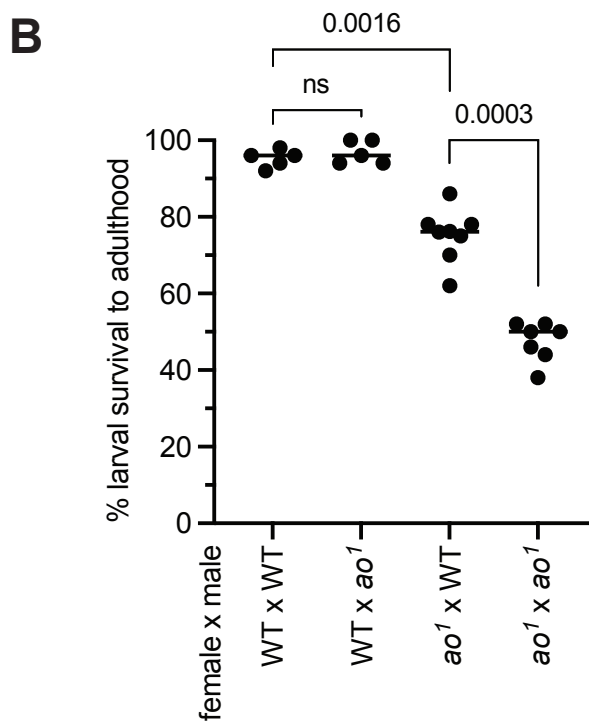

**Figure S4**

Supplement: iyag036_Supplementary_Data [file iyag036_supplementary_data.zip › Supplemental_Figure_S4_GENETICS-2025-308878.pdf]

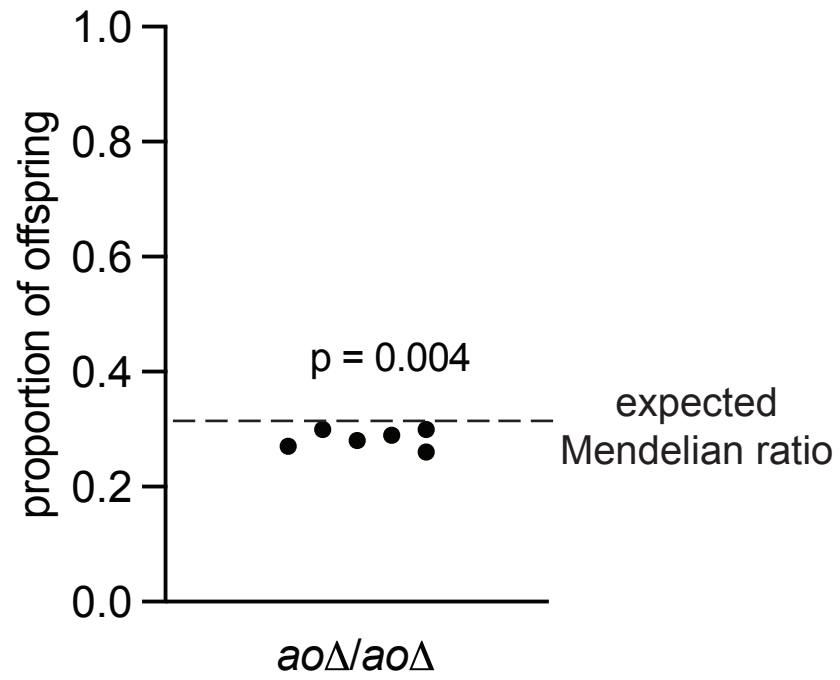

**Figure S5**

Supplement: iyag036_Supplementary_Data [file iyag036_supplementary_data.zip › Supplemental_Figure_S5_GENETICS-2025-308878.pdf]

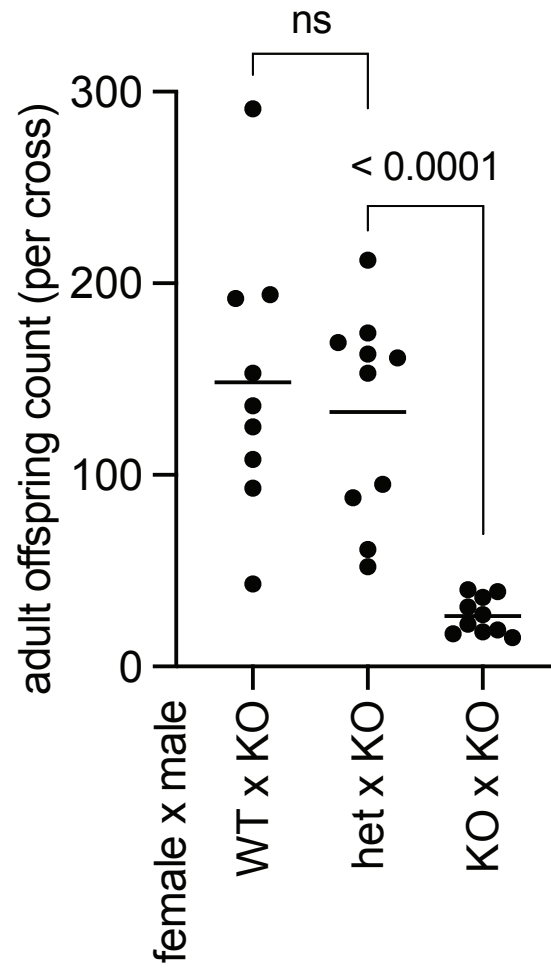

**Figure S6**

Supplement: iyag036_Supplementary_Data [file iyag036_supplementary_data.zip › Supplemental_Figure_S6_GENETICS-2025-308878.pdf]

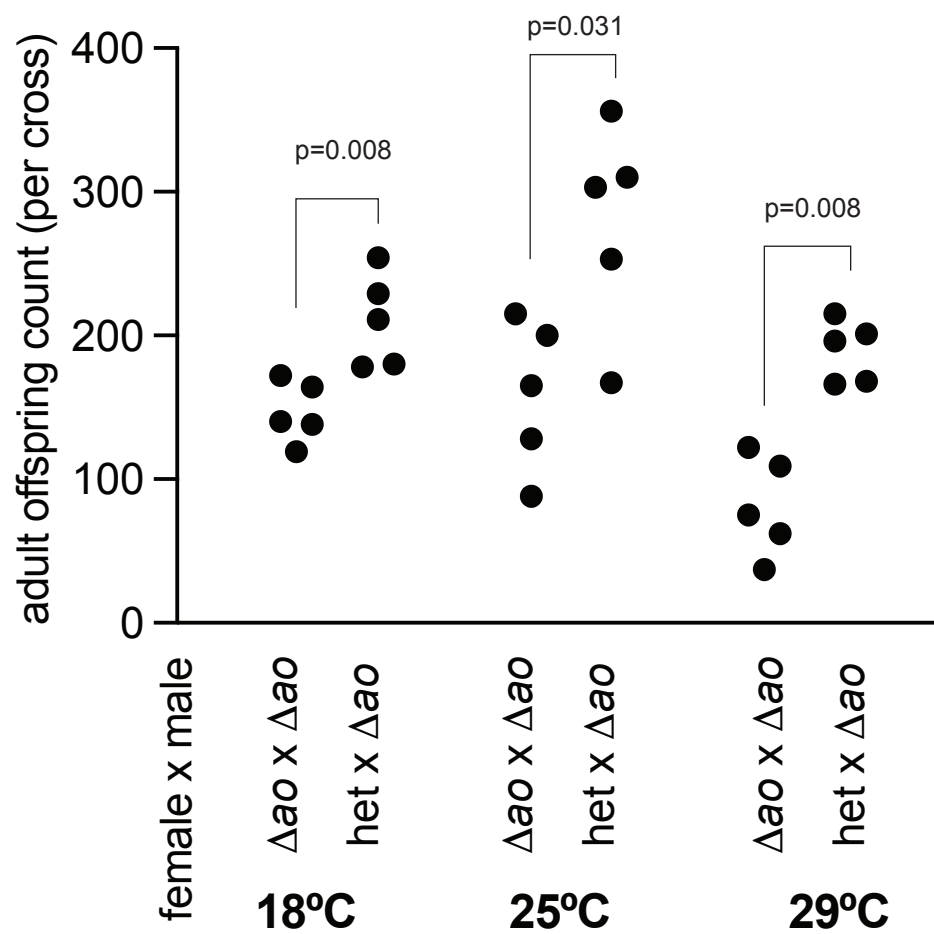

**Figure S7**

Supplement: iyag036_Supplementary_Data [file iyag036_supplementary_data.zip › Supplemental_Figure_S7_GENETICS-2025-308878.pdf]

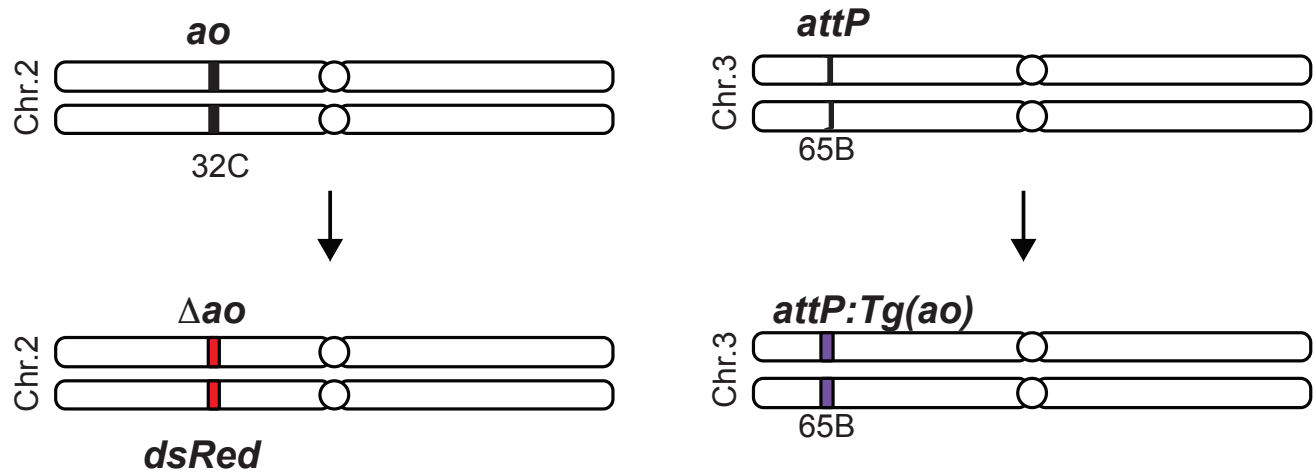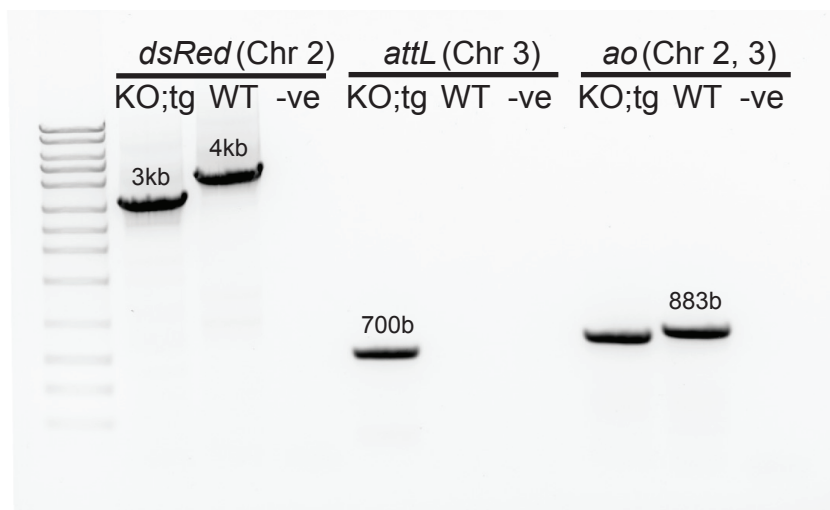

**Figure S8**

Supplement: iyag036_Supplementary_Data [file iyag036_supplementary_data.zip › Supplemental_Figure_S8_GENETICS-2025-308878.pdf]

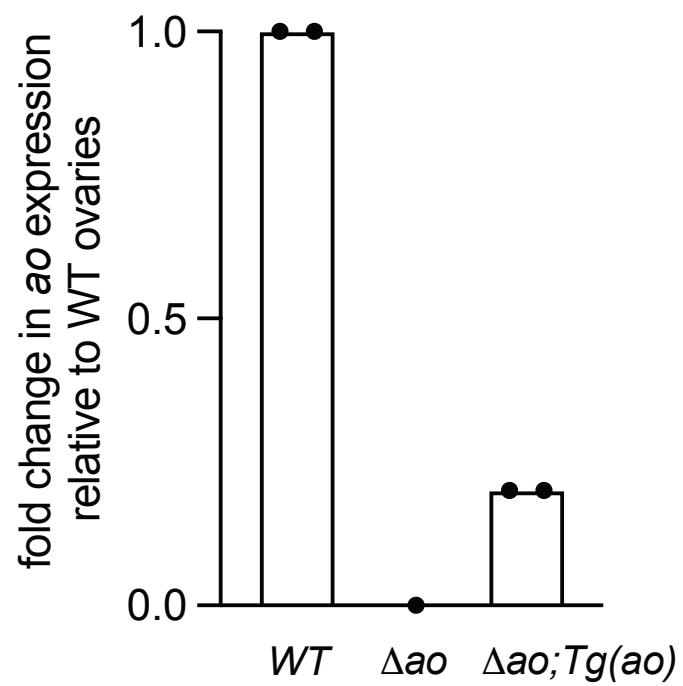

**Figure S9**

Supplement: iyag036_Supplementary_Data [file iyag036_supplementary_data.zip › Supplemental_Figure_S9_GENETICS-2025-308878.pdf]
